# A Double-Edged Sword: The Anti-Cancer Effects of Emodin by Inhibiting the Redox-Protective Protein MTH1 and Augmenting ROS in NSCLC

**DOI:** 10.7150/jca.41160

**Published:** 2021-01-01

**Authors:** Divya Wahi, Deepika Soni, Abhinav Grover

**Affiliations:** School of Biotechnology, Jawaharlal Nehru University, New Delhi, India - 110067.

**Keywords:** lung cancer, NCSLC, MTH-1, NUDT-1, ROS, Emodin, oxidative DNA damage

## Abstract

**Background:** Reactive oxygen species (ROS), playing a two-fold role in tumorigenesis, are responsible for tumor formation and progression through the induction of genome instability and pro-oncogenic signaling. The same ROS is toxic to cancer cells at higher levels, oxidizing free nucleotide precursors (dNTPs) as well as damaging DNA leading to cell senescence. Research has highlighted the tumor cell-specific expression of a redox-protective phosphatase, MutT homolog 1 (MTH1), that performs the enzymatic conversion of oxidized nucleotides (like 8-oxo-dGTP) to their corresponding monophosphates, up-regulated in numerous cancers, circumventing their misincorporation into the genomic DNA and preventing damage and cell death.

**Methods:** To identify novel natural small molecular inhibitors of MTH1 to be used as cancer therapeutic agents, molecular screening for MTH1 active site binders was performed from natural small molecular libraries. Emodin was identified as a lead compound for MTH1 active site functional inhibition and its action on MTH1 inhibition was validated on non-small cell lung cancer cellular models (NSCLC).

**Results:** Our study provides strong evidence that emodin mediated MTH1 inhibition impaired NSCLC cell growth, inducing senescence. Emodin treatment enhanced the cellular ROS burdens, on one hand, damaged dNTP pools and inhibited MTH1 function on the other. Our work on emodin indicates that ROS is the key driver of cancer cell-specific increased DNA damage and apoptosis upon MTH1 inhibition. Consequently, we observed a time-dependent increase in NSCL cancer cell susceptibility to oxidative stress with emodin treatment.

**Conclusions:** Based on our data, the anti-cancer effects of emodin as an MTH1 inhibitor have clinical potential as a single agent capable of functioning as a ROS inducer and simultaneous blocker of dNTP pool sanitation in the treatment of NSCL cancers. Collectively, our results have identified for the first time that the potential molecular mechanism of emodin function, increasing DNA damage and apoptosis in cancer cells, is via MTH1 inhibition.

## Introduction

Currently, worldwide statistics reveal lung carcinoma is the chief reason for cancer related deaths 1. The most widespread type of lung cancer, non-small cell lung cancer (NSCLC) is difficult to treat owing to its relative insensitivity to chemotherapeutics and radiotherapy and its low five-year survival rate 2, 3. Despite several successful advances on the mechanistic insights into lung cancer development, the concise molecular pathways causing mutations leading to carcinogenesis are still being elucidated. Studies note that cellular environment stresses, such as reactive oxygen species (ROS) causing oxidative stress, are key contributors to lung cancer pathogenesis 4-6. In addition to the generation of ROS as a routine by-product of the respiratory chain's cellular metabolic process, certain exposures of the cellular environment, such as tobacco smoke, are responsible for the increase in ROS levels of normal cells 4, 7.

ROS is responsible for the oxidation of cellular pools of nucleotide precursors and protein building blocks, inducing multilevel genetic mutations leading to cell senescence 8. Causing the oxidation of precursor nucleotide moieties and the nucleic acid material itself, ROS leads to the production of 8-oxo-guanine (8-oxoG) in the nucleotide pool via direct oxidation of dGTP or DNA guanine bases. Then the DNA polymerases often insert oxidized 8-oxo-dGTP that pair opposite to the adenine or cytosine bases during DNA replication, causing an oxidized base lesion of G to T transversional mutations 9.

The gradual cellular accumulation of such nucleic material lesions induces increased cellular dysfunction, spontaneous tumorigenesis susceptibility, and eventually carcinogenesis. To tackle such deleterious cellular outcomes, human cells equip themselves with enzymes to sanitize the oxidized nucleotide pools. The 18kD human MutT homologue 1 (MTH1) protein, also known as the Nudix hydrolase 1 (NUDT1), is the predominant pyrophosphatase that functions to hydrolyze the oxidized purine nucleotide bases at the cellular level leading to their degradation 10, 11. MTH1 hydrolyses the oxidized purine deoxyribonucleotide bases such as 8-oxo-dGTP, 2-OH-dATP, and 8-oxo-dATP, along with their corresponding ribonucleotide analogues to their monophosphate equivalent forms 12. This enzymatic function of MTH1 protein prevents the incorporation of oxidized nucleotide precursors into the rapidly proliferating genetic material 13, 14.

Although MTH1 has a crucial cellular role, germline MTH1 knockout mice are developmentally normal and the treatment of such mice with ROS generating chemicals increases DNA oxidation and cellular toxicity 15-17. This implies MTH1 genetic knockdowns and small molecule inhibition leads to tumor specific DNA damage and senescence both at the *in vitro* and *in vivo* levels, while showing minimal outcomes on the normal cells having lower ROS levels. This research emphasizes that the MTH1 function becomes significant in biological conditions where sustaining elevated levels of ROS increases the oxidized nucleotide pool burdens. Thus, MTH1 function becomes crucial to cellular environments with increased ROS.

As studies have stated MTH1 is abundantly expressed in numerous cancer types, such as lung, brain, and gastric cancers. They are regularly exposed to increased levels of oxidative stress compared to the adjacent normal cells, indicating there is a significant MTH1 contribution to tumor cell proliferation 18-20. Research indicates MTH1 is indeed selectively essential in NSCLC cells to support uninterrupted cell proliferation and maintain genome integrity 21. Also, xenograft studies show that MTH1 inhibition resulted in decreased tumor proliferation 22, 23. MTH1 mRNA overexpression is observed in cancer cells and has an inverse correlation with 8-oxo-dG levels in the cellular milieu 18. Researchers have also observed an increased MTH1 function in NSCLC in comparison to normal lung tissue. Additionally, the blocking of MTH1 function contributes to epithelial-mesenchymal transition and cell proliferation suppression, revealing MTH1 as a promising therapeutic target, particularly in NSCLC cases 24, 25.

Owing to their rapid proliferation and growth, cancer cells tend to re-organize their cellular metabolic pathways. Consequently, they are under continuous exposure to elevated ROS levels leading to 8-oxo-GTP accumulation and eventual cell death 26, 27. Studies state robust MTH1 functionality is essential for avoiding the oxidative stress laden hypermetabolic tumors that undergo rapid cell proliferation 28. Thus, cancer cells gain function, acquiring specific mechanisms that prevent 8-oxoG base accumulation in the DNA. The significance of MTH1 enzyme function in cancer cell survival is currently a highly controversial topic and the focus of intense research. Recently researchers have identified chemical inhibitors to MTH1 that show anti-cancer function both *in vitro* and *in vivo* 22, 23. However, the identification of MTH1 inhibitors of natural origin has yet to be accomplished. The rising need for therapeutic interventions in cancer progression is an emerging field in cancer management via the identification of phytochemicals that are functionally non-toxic, cost-effective as drug candidates, and physiologically bioavailable in the organism. In the present study, we screened MTH1 inhibitors from natural small molecule compound libraries and identified emodin as a leading novel natural MTH1 inhibitor. We also highlight the dual function of emodin leading to cancer cell senescence; it can cause increased ROS in NSCLC cells along with MTH1 function inhibition. These findings are in line with previous works, indicating emodin causes increased ROS in cancer cells but the exact mechanism of how ROS augmentation led to apoptosis in cancers remained un-elucidated until now 29-31.

Emodin (1, 3, 8-trihydroxy-6-methylanthraquinone) is an ancient natural hydroxy-anthraquinone that is isolated from the roots and rhizomes of a number of medicinal plants (frangula, rhubarb, etc.) and exhibits diverse biological effects such as being antibacterial, anti-inflammatory, immunosuppressive, vasorelaxant, and diuretic 32-36. Recently, emodin gained special attention as *in vitro* and *in vivo* research stressed its anti-cancer effects through multiple mechanisms in several types of malignancies by inhibiting cancer cell growth, increasing ROS levels, chemotherapeutic sensitization, and apoptosis induction 29, 37-40. The existing literature on emodin has primarily focused on the direct toxicity it exerts on cancer; however the precise underlying molecular pathway is still to be elucidated.

In previously conducted studies, it has been reported that emodin exerts apoptotic effects on various human cancer cell lines, including NSCLC subtypes, but the effects of emodin on NCI-H-520 cells has remained unexplored. Thus, our research examines for the first time the effects of emodin on the NCI-H-520 cell line model. The recent resurgence of research on the anti-cancer potential of emodin has shown great advances and is found to have anti-tumor effects on various types of cancers with diverse mechanisms. Thus, the exact mechanism of emodin requires further study.

As per our extensive search, the current study is the first to demonstrate the association between emodin causing increased ROS and simultaneous MTH1 functional inhibition, acting as a double-edged sword against cancer. We screened MTH1 inhibitors from natural small molecule compound libraries and identified emodin as a lead novel natural MTH1 inhibitor. Although some works state emodin treatment has been responsible for increased ROS and subsequent DNA damage, this is the first study to directly show a correlation between emodin mediated MTH1 activity inhibition using molecular docking techniques and biophysical analysis; this leads to increasing DNA damage and apoptosis in cancers. The present study highlights the combined effects of increasing ROS and the simultaneous blocking of dNTP pool sanitation by MTH1 on human non-small cell lung cancer cell line models.

## Methods

### Materials

Roswell Park Memorial Institute (RPMI) medium containing phenol red, fetal bovine serum (FBS), and penicillin-streptomycin antibiotics (200 units/ml) were obtained from Gibco BRL (Grand Island, NY, USA). The 96-, 24-, and 6-well plates and T_25_ and T_75_ cell culture flasks were obtained from NUNC (Roskilde, Denmark). Dimethyl sulfoxide (DMSO) was procured from Merck-Millipore (NJ, USA). The emodin (E7881), and 3-(4, 5-dimethylthiazol-2-yl)-2, and 5-diphenyltetrazolium bromide (MTT) were procured from Sigma Aldrich (USA). The JC-1 iodide (sc-364116), 3,3′-dihexyloxacarbocyanine iodide (DIOC_6_) (sc-205905), and propidium iodide (sc-3541) were procured from Santa Cruz Biotech (Santa Cruz, CA, USA). The CellROX™ Green (C10444), CellROX™ Deep Red (C10422), and Hoechst 33342 (H1399) were procured from Thermo Fisher Scientific (USA). The organic solvents were of HPLC-grade (Merck, NJ, USA). All other chemicals were from Sigma Aldrich (USA) or as otherwise specified. The list of antibodies used is provided in the supplementary data.

### Cells and Cell Culture

The NSCLC cell line models NCI-H-520 (squamous cell carcinoma), NCI-H-460 (large cell carcinoma), and A-549 (adenocarcinoma) cells were obtained from the cell repository at National Centre for Cell Science (NCCS, Pune, India). All cells were cultured in RPMI-1640 media (Gibco) supplemented with 10% (v/v) fetal bovine serum (Gibco), 100 μg/ml streptomycin, and 100 U/ml penicillin (Gibco). The cultures were grown and maintained at 37°C in a humidified incubator containing 5% CO_2_. The cultures were passaged on reaching 80% confluence and split into a ratio of 1:4 to be used for experiments.

### Cell Cycle Analysis

The NSCLC cells were cultured until 80% confluence, and then seeded in 6-well plates in 3 ml of the RPMI medium at a cell density of approximately 10^5^ cells/ml. The cells were subsequently treated with emodin at different dilutions (0, 25, 50, and 75 μM) in DMSO, (vehicle < 0.5% volume) for 24, 48, and 72 h. Post-treatment, the cells were harvested by trypsinization and washed with PBS. The cells were then fixed using ice-cold 70% ethanol for 24 h at 4°C. The samples were then treated with 20 μg/ml RNase A and 50 μg/ml PI (propidium iodide) for 30 min. Then, the percent cell populations in the G0/G1, S, and G2/M phases were acquired by flow cytometry (BD FACSAria Flow Cytometer, Becton Dickinson Biosciences, USA). A small volume of cells without drug treatment were used as an unstained control.

### Mitochondrial Membrane Potential (ΔΨM) Assay

#### JC-1 and DIOC_6_ 3 Iodide: Fluorometric Assessment of Mitochondrial Membrane Potential

The NSCLC cells were cultured until 80% confluence, and then seeded in 96-well plates in 100 μL of the RPMI medium at a cell density of approximately 10^3^ cells/well. The cells were treated with emodin at different dilutions ( 0, 1, 10, 25, 50, 75, 100, and 200 μM ) in DMSO (vehicle < 0.5% volume) for 24, 48, and 72 h. Post-treatment, the media was aspirated and 100 µl of fresh media containing 1µg/ml JC-1 (working concentration from 1mg/ml JC-1 in DMSO stock solution) or 1µM DIOC6 3 iodide (working concentration from 1mM DIOC6 3 iodide in DMSO stock solution) was added to each well. The 96-well plate was incubated at 37°C in a humidified 5% CO2 chamber for 20 min. After incubation, the JC-1 dye fluorescence was acquired with a 96-well ELISA plate-reader (2300 EnSpire Multimode Plate Reader, PerkinElmer, MA, USA) using wavelengths of excitation at 488 nm, emission at 530 nm for monomers and excitation at 540 nm, and emission at 590 nm for J-aggregates.

### Annexin V/PI Apoptosis Analysis

#### Flow Cytometric Assessment of Apoptosis

The NSCLC cells were cultured until 80% confluence, and then seeded in 6-well plates in 3 ml of the RPMI medium at a cell density of approximately 10^5^ cells/ml. The cells were subsequently treated with emodin at different dilutions (0, 25, 50, and 75 μM) in DMSO (vehicle < 0.5% volume) for 24, 48, and 72 h. Post-treatment, the cells were harvested by trypsinization and washed with PBS. The cells were then stained with 5 µL Alexa Fluor® 488 annexin V and 1 µL 100 µg/mL propidium iodide (PI) working solution per 100 µL cell sample in the dark at room temperature for 15 min, according to the manufacturer's instructions (Dead Cell Apoptosis Kit with Annexin V Alexa Fluor™ 488 & PI, Thermo Fisher Scientific, USA). After incubation the cells were kept on ice. Immediately, the apoptotic cells were determined by flow cytometry (BD FACSAria Flow Cytometer, Becton Dickinson Biosciences, USA) acquiring fluorescence emission at 530 nm and >575 nm. A small volume of cells without drug treatment were used as an unstained control.

### Western Blot

The proteins of the total cell lysate were estimated using the Pierce™ BCA Protein Assay Kit (Thermo Fisher Scientific, USA). Equal volumes of protein were loaded in each well and separated on 6%-12% SDS-PAGE gels using the protein electrophoresis Western blotting assembly (Bio-Rad, Hercules, CA, USA) according to the manufacturer's instructions. The proteins were electrophoresed at 60V in a stacking gel and 100 V in a resolving gel. After separation, they were transferred onto nitrocellulose membranes (Merck-Millipore, NJ, USA) followed by blocking with 3% skimmed milk in tris buffered saline-0.1% Tween-20 (TBS-T) at room temperature for 2 h. The blots were probed with different primary antibodies at their specific dilutions (1:200-1:1000 in 3% BSA in TBS-T) at 4°C overnight and then washed in TBS-T. Horseradish peroxidase-conjugated secondary antibodies (1:2000) at room temperature for 1 h and then developed using the Immobilon Western Chemiluminescent HRP Substrate (Merck-Millipore, NJ, USA).

### Modified Alkaline Comet Assay

Alkaline single-cell gel electrophoresis was performed on NSCLC cells to assess the DNA damaging potential of emodin. The cells were seeded in 6-well plates at a concentration of approximately 10^5^ cells/well and treated with emodin at different dilutions (0, 25, 50, and 75 μM) in DMSO (vehicle < 0.5% volume) for 24, 48, and 72 h. After treatment, the cells were centrifuged, harvested, and washed with PBS. Immediately, they were resuspended in a volume of 100 μL low-melting agarose (LMA) and spread on the surface of frosted slides coated with 1% normal melting agar (NMA) and a coverslip was placed on top of the mixture. The slides were kept on ice for 10 min before removing the coverslips. The cells were lysed by incubating the slides in a lysis buffer (2.5 M NaCl, 0.1 M EDTA, 10 mM Tris, pH 10, 1% Triton X-100, 10% DMSO) for 1 h and treated with an alkaline buffer (0.3 M NaCl, 1 mM EDTA) for 40 min in the dark. The slides were then immersed in the electrophoretic buffer (300 mM NaOH, 1 mM EDTA), electrophoresed at 22 V and 300 mA for 30 min and immediately neutralized using the neutralization buffer (0.4M Tris-HCl, pH 7.5). The slides were stained with ethidium bromide and analyzed on a Nikon real time laser scanning confocal microscope at 60X and 20X magnification with supporting software. A set of 100 comets were analyzed in triplicate from each sample set, including the vehicle control. The tail moment was then calculated as % DNA in the comet tail × tail length, using Andor Komet version 7.1 software.

### Terminal Transferase dUTP Nick End Labeling (TUNEL) Assay

For confocal microscopy based determination of cells exhibiting DNA fragmentation using TUNEL assay (according to a modified version of the manufacturer's recommended protocol, DeadEnd™ Fluorometric TUNEL System Kit, Promega, WI, USA), the NSCLC cells were cultured until 80% confluence and seeded on coverslips, placed in 24-well plates at a cell density of approximately 10^4^ cells/well. The cells were treated with emodin at different dilutions (0, 25, 50, and 75 μM) in DMSO (vehicle < 0.5% volume) for 24, 48, and 72 h; following which the cells were washed (twice) with PBS and fixed with 4% paraformaldehyde in PBS for 20 min. The fixed cells were then rinsed with PBS and permeabilized using 0.5% TritonX-100 for 5 min.

The PBS washed slides were incubated for 10 min with an equilibration buffer. The cells on the coverslips were subsequently incubated with the incubation buffer having a mix of the equilibration buffer, nucleotide mix, and rTdT enzyme in each well, in a dark humidified chamber at 37°C for 1 h. Post-incubation, the cells were treated with 2X SSC buffer for 15 min at room temperature to stop the reaction and a PBS wash was performed. The DNA was counterstained with a 2 µg/ml working Hoechst staining solution from a Hoechst 10 mg/mL stock in PBS for 10 min at room temperature and the coverslips were mounted in DABCO (Sigma Aldrich) mounting medium (containing 1% DABCO [w/v] in 80% glycerol and 20% PBS). The cells were visualized using a Nikon real time laser scanning confocal microscope at 60X magnification with supporting software.

### Surface Plasmon Resonance (SPR) Analysis

Using a monolayer surface of self-assembled 11-mercaptoundecanoic acid (MUA) on a gold surface, the Autolab Esprit Instrument (Eco Chemie BV, Netherlands) was activated with N-hydroxysuccinimide (NHS, 0.05 M)/N-ethyl-N-(diethylaminopropyl) carbodiimide (EDC, 0.2 M) for 600 seconds. Subsequently, MTH1 as a ligand was immobilized to the activated sensor surface for 600 seconds at a concentration of 30 μg/ml and dissolved in the immobilization buffer (20 mM sodium acetate buffer [pH 5.0], 0.22 μm filtered and degassed). After MTH1 ligand immobilization, the gold surface was blocked for 200 seconds with 100 mM ethanolamine (pH 8.5), followed by regeneration for 300 seconds using 50 mM NaOH (0.22 μm filtered and degassed). Then, the kinetic analysis was performed by flowing different concentrations of the analyte emodin (1 mM to 25 mM) on the immobilized protein MTH1 in a running buffer (20 mM sodium phosphate buffer (pH 7.4), 0.22 μm filtered and degassed) at 25°C. The parameters of association kinetics were monitored for 300 seconds followed by dissociation kinetics for 100 seconds. After each cycle of association and dissociation the binding surface was regenerated using 50 mM NaOH and a baseline of 120 seconds was run. All the binding kinetic experiments were performed in triplicate on three separate sensor chips on the Autolab SPR apparatus using emodin as an analyte and (S)-crizotinib as the positive control. The results in the form of reference-subtracted sensorgrams were analyzed using the Autolab equipment software. The binding constant K_D_ was calculated as K_D_/K_A_ and plotted as a differential response of emodin binding to MTH1.

### 8-OHdG estimation

The 8-OHdG DNA was extracted from NSCLC cells using the commercial kit 8-oxo-dG ELISA kit (R&D Systems, Inc. MN, USA) according to the manufacturer's instructions. The absorbance was read at 450 nm.

### Densitometry and Statistical Analysis

The western blotting bands were scanned using the Syngene G:Box Gel Doc system and the mean density was analyzed using ImageJ software (version 1.48v, NIH, USA). Densitometry data is represented as “fold change” as compared with respective vehicle DMSO control, after normalization with respective loading controls (β-actin).

All measurements were carried out in triplicate, with error bars representing the respective standard deviation occurring at each data point. The significance of all cases was verified using a two-tailed *t*-test. The *p*-value notations were reported as *p < 0.05, **p < 0.01, and ***p < 0.001.

Detailed procedures for Screening of MTH-1 inhibitors, Molecular dynamics, Assessment of cell morphology, Assay of cell growth and Viability, Wound-healing Assay, Mitochondrial membrane potential (ΔΨM) assay, Nuclear morphological Assessment, Anenexin V/PI Apoptosis Analysis, Cell lysate Preparation, Measurement of intracellular ROS, DNA Fragmentation Analysis, Indirect Immunofluorescence, Bacterial Expression and purification of MTH-1, Circular Dichroism Spectroscopy analyses are provided in the supplementary data.

## Results

### Virtual Screening and Docking Studies to Identify MTH1 Binding Compounds

A virtual screening of the Sigma natural compound database and the Natural Remedies natural compound database against MTH1 protein was carried out. The active site of MTH1 protein is composed of the residues Leu9, Phe27, Phe72, Met81, Val83, Trp117, Trp123, and Phe139 41. To screen for MTH1 abrogators, the active site residues along with Asn33, Asp119, and Asp120 were used for grid generation 41, 42.

**Figure 1:**
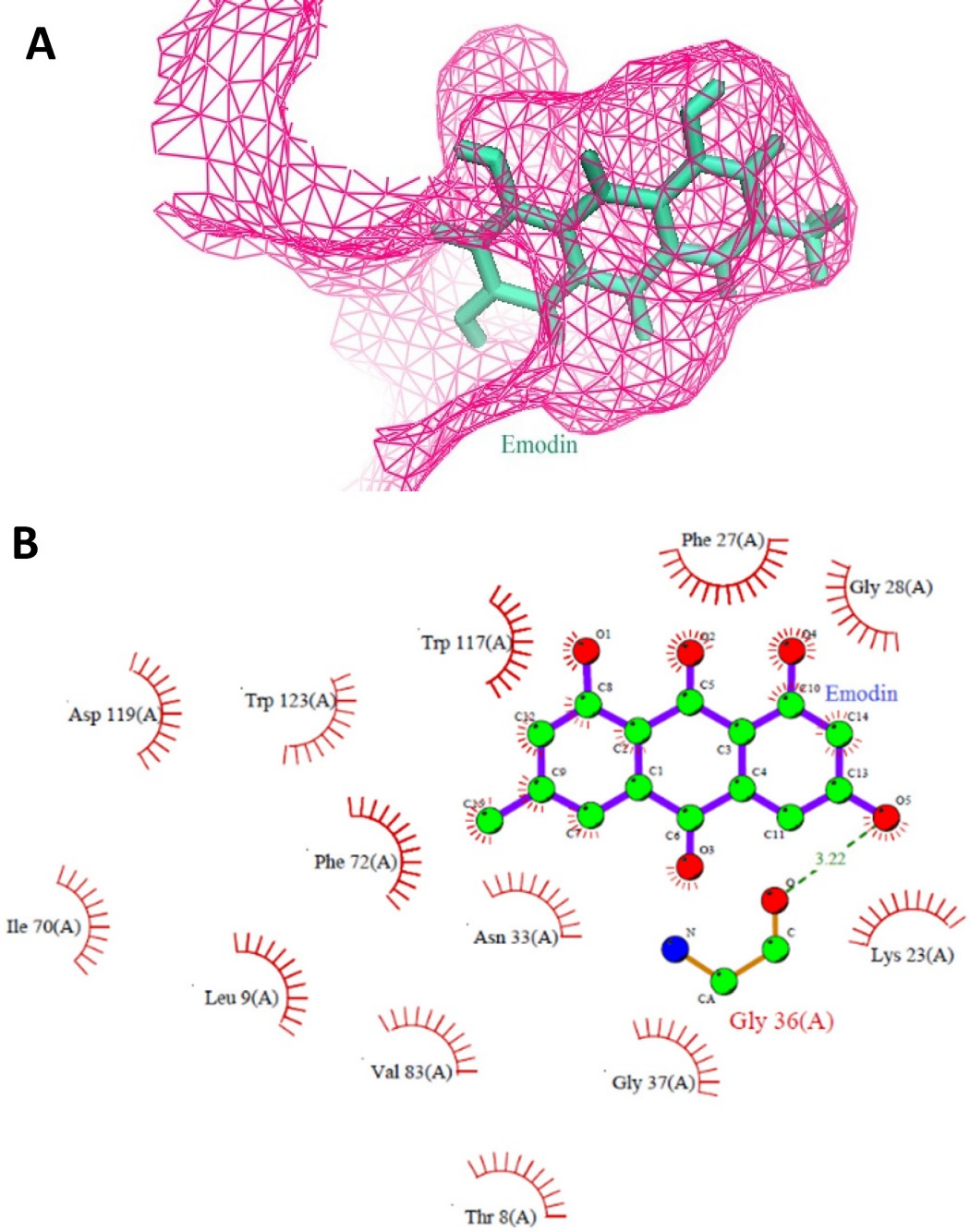
** A.** Stereo view of the MTH1 binding site is shown. The protein portions building up the binding site are coloured as pink mesh and emodin as ligand (Green) is embedded deep in the active site of MTH1. **B.** Molecular interactions between MTH1 protein and emodin, showing the hydrophobic interactions (in red) and hydrogen bonds (in green). The diagram is illustrated using LIGPLOT software.

The top results based on Glide HTVS docking scores, fulfilling Lipinski's rule, were selected for next level filtering and screening using the stringent docking protocol XP. All the compounds that achieved Glide HTVS scores greater than -7 Kcal/mol were subjected to XP docking, after which the top three natural compounds showing the highest affinity interactions with MTH1 (ligand_71308639 taxifolin hydrate [Sigma], ligand_148 quercetin dihydrate [Natural Remedies], and ligand_3220 emodin [Sigma]) were selected. Taxifolin hydrate, quercetin dihydrate and emodin had strong binding interactions with MTH1 as shown by their respective Glide scores of -8.76 Kcal/mol, -8.74 Kcal/mol, and -7.63 Kcal/mol, respectively (Figure 1). All three natural compounds occupied the targeted active site in the MTH1 structure blocking the oxidized dNTPs' access into the MTH1 cavity for interaction. Table 1 depicts the docking result in terms of the Glide score from XP, the Glide energy, the major interacting residues, and the H-bond distances formed.

However, after reconfirmation of the docking scores with the AutoDock version 4.0.1 software 43, emodin was found to be the most stable binding partner at the MTH1 active site. The docking scores were -6.83 Kcal/mol for Emodin, -5.77 Kcal/mol for taxifolin hydrate, and -5.81 Kcal/mol for quercetin dihydrate (Table 2). Further, studies in the literature noted previous links between ROS, DNA damage, and emodin 29, 38, 44-46; thus emodin was investigated as the lead small molecule natural compound for MTH1 inhibition studies.

### Specificity of Emodin to MTH-1 and Binding Mode Analysis

**Figure 2:**
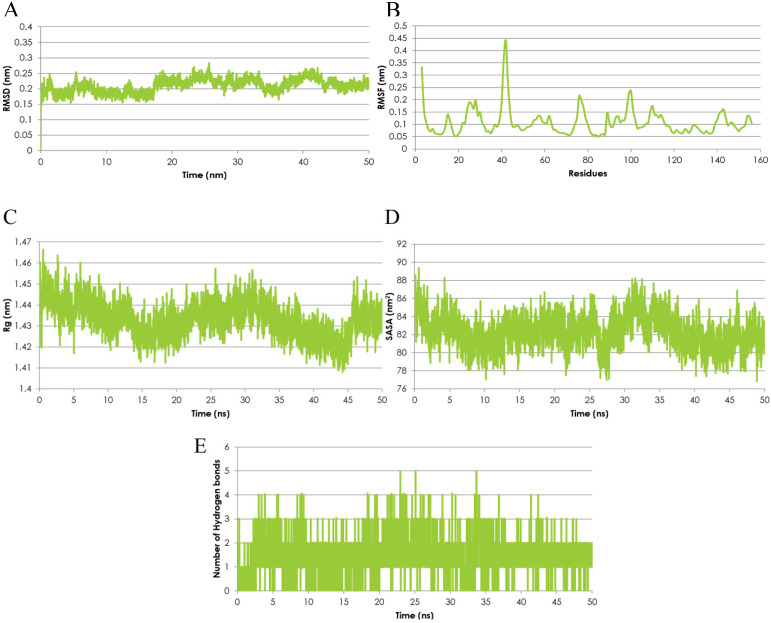
Emodin-MTH1 complex - Graphical representation of **A.** The RMSD analysis of the 50 ns trajectory to examine the detailed dynamics of all binding atoms. **B.** The RMSF analysis. **C.** The radius of gyration (Rg) analysis. **D.** The SASA analysis. **E.** The H-bond lengths of the simulated complex.

The Molecular Dynamics (MD) simulation was performed using the emodin-MTH1 complex docked conformation to analyze the complex conformational dynamics. The MD trajectory frames of the protein-drug complex were obtained for a 50 ns time scale using the cluster analysis technique and plotted for post-simulation interactions. The root mean square fluctuation (RMSD) analysis of the 50 ns trajectory shows an increased RMSD value (> 2nm) after ~18.5 ns but the deviations remain in the overall range of 0.1 nm until the end of the simulations. Thus, the RMSD profile was consistently below 0.3 nm for the entire duration of the simulations and in a time span of 50 ns the emodin-MTH1 complex trajectory achieved convergence (Figure 2A). The mean residue flexibility of the emodin-MTH1 complex structure was also investigated using the RMSF analysis. The RMSF score of the emodin-MTH1 complex had a peak of 0.45 nm for the residues 37 to 44 (Figure 2B).

The statistical analysis of the radius of gyration (Rg) for the emodin-MTH1 complex through the 50 ns trajectory indicated there is an increased characteristic stability over time to a final 1.41 nm Rg value as compared to the initial 1.46 nm Rg (Figure 2C). The slight decrease in the Rg score can be interpreted as greater compactness and folding of the protein to make the drug more available for the initiation of a stable interaction and binding function. The solvent-accessible surface area (SASA) of the emodin-MTH1 complex stabilizes over a simulation time of 50 ns (Figure 2D). Decreased SASA values indicate the increasing hydrophobic surface of the protein highlight there is an increasing fold pattern and relative stabilization of the emodin-MTH1 structure and decreased intrinsic flexibility, increasing the likelihood of stable binding of emodin with MTH1.

The emodin-MTH1 structural interactions and intermolecular H-bonding pattern was studied and the complex stability was described for a simulation duration of 50 ns. The H-bond lengths decreased with an increase in the simulation time, making the complex more stable, while initially the H-bond interacting residues had greater bond lengths and weaker interactions (Figure 2E). Similar to the conclusions from the RMSF analysis, the simulated emodin-MTH1 complex shows lower flexibility of the conformation and higher stability owing to the reduced H-bond interactions. Conclusively, the post MD simulation interaction study highlights the increased stability of the simulated complex in contrast to the initial complex. The values of the SASA energies are calculated in Table 3.

### The Sensitivity of NSCLC Cell Lines to Emodin

To further validate the above molecular dynamic conclusions of docking emodin with MTH1 protein, human NSCLC cell lines NCI-H-520 (squamous cell carcinoma), NCI-H-460 (large cell carcinoma), and A-549 (adenocarcinoma) were treated with emodin (25, 50 and 75 μM) or vehicle (DMSO < 0.5% volume) for 24, 48, and 72 h. The overall morphological assessment of cell health and growth was performed visually and photographed. There was a marked decrease in cell number and cell growth in a dose and time dependent manner, as the cells appeared to be more rounded and less adherent to the surface of the flask in the microscope (Supplementary Figure 1).

**Figure 3:**
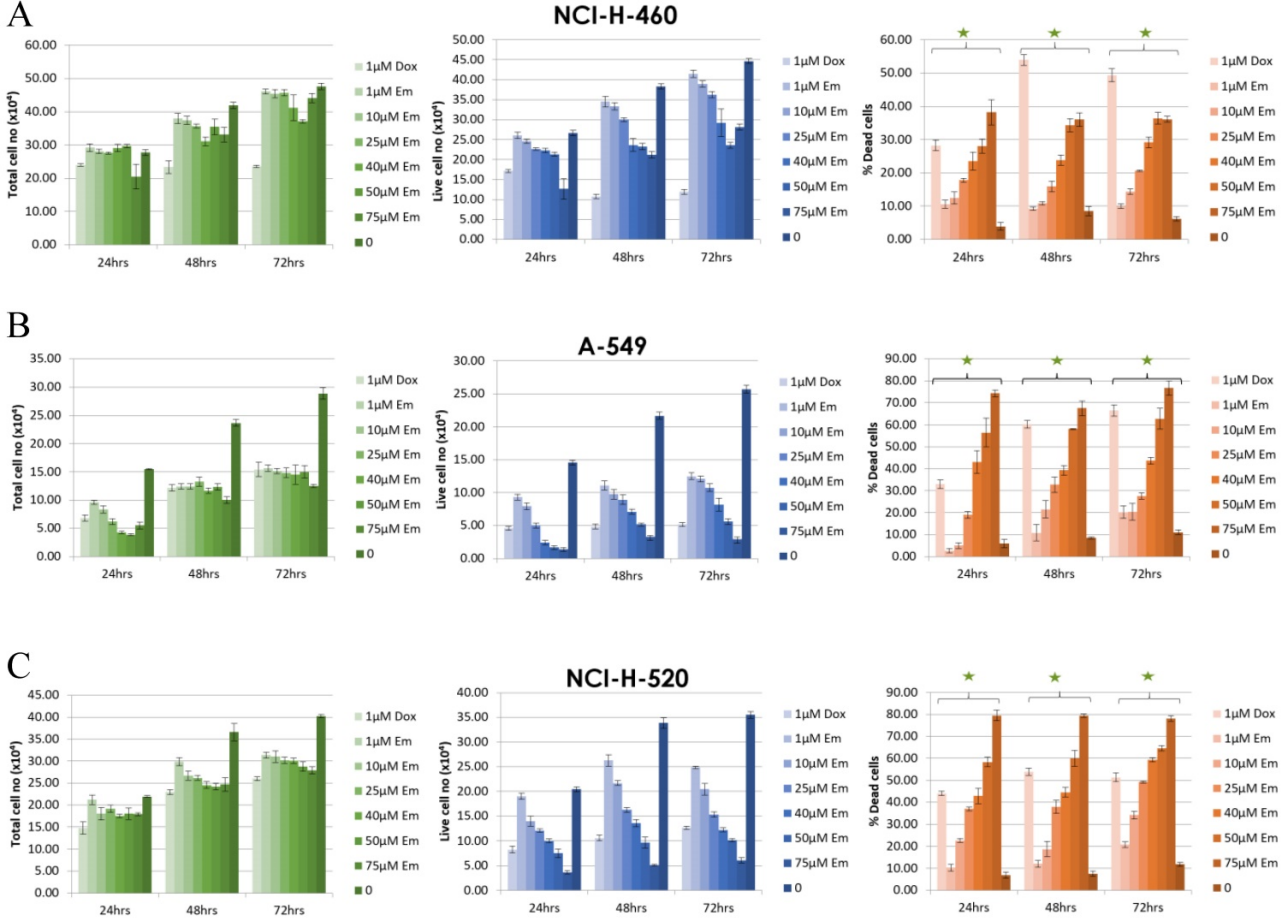
** Measurement of cell proliferation by Trypan Blue assay. A**, **B** and **C** are graphical representation of the Total, Live and Percent Dead cells caused by increasing emodin treatment (0, 1, 10, 25, 40, 50 and 75 µM) at 24, 48 and 72 h, as measured by phase-contrast for NCI-H-460 (Large cell carcinoma), A-549 (adenocarcinoma) and NCI-H-520 (squamous cell carcinoma) cell lines respectively. Data shown as mean ± S.E.M. of three independent experiments and * indicate *P* values < 0.05.

The effect of emodin on cell proliferation was then assayed using the trypan blue dye exclusion assay. The NSCLC cells showed dose and time dependent growth inhibition. In the NCI-H-460 cells, the total cell number decreased by 77% (*p <* 0.05) to 62% (*p <* 0.001) after 48 h and 63% (*p <* 0.001) to 46% (*p <* 0.001) following 72 h of emodin treatment compared to 24 h (Figure 3A). In the A-549 cells, the same emodin concentrations effectively decreased the total cell numbers by 77% (*p <* 0.05) to 55% (*p <* 0.001), 61% (*p <* 0.05) to 44% (p<0.001) following 48 and 72 h of emodin treatment compared to 24 h (Figure 3B). In the NCI-H-520 cells, the range of decrease in total cell numbers was 71% (*p <* 0.05) to 60% (*p <* 0.001) and 68% (*p <* 0.05) to 54% (*p <* 0.001) following 48 and 72 h of emodin treatment with respect to 24 h (Figure 3C). We observed a linear increase in the percentage of dead cells along with the increasing concentrations of emodin with time in all the NSCLC cells, which was moderate but significant at higher doses (Figure 3).

**Figure 4:**
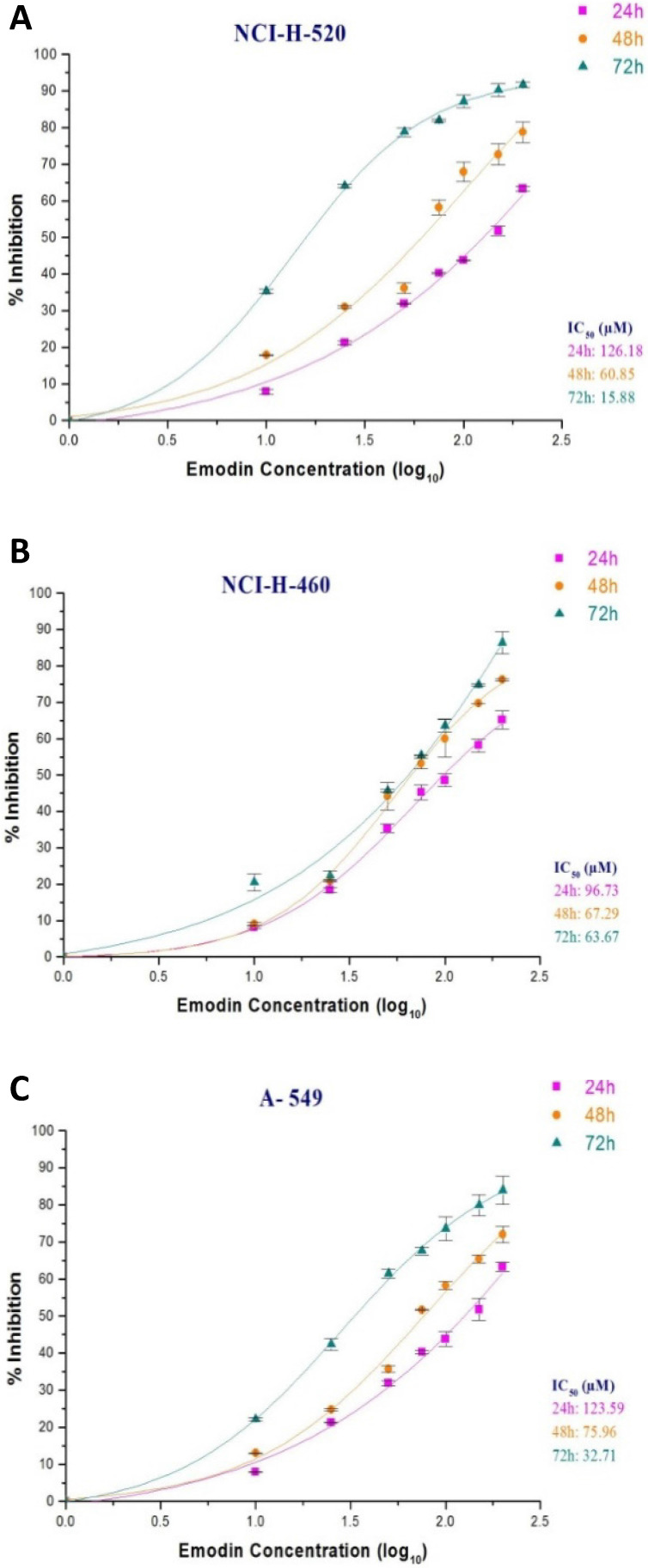
** Measurement of cell proliferation by MTT assay. A**, **B** and **C** are graphical representation of percent inhibition caused by increasing emodin treatment (0, 10, 25, 50, 75, 100, 150 and 200 µM) at 24, 48 and 72 h, for NCI-H-520 (squamous cell carcinoma), NCI-H-460 (Large cell carcinoma) and A-549 (adenocarcinoma) cell lines respectively as measured by MTT colorimetric assay, absorbance taken at 595nm. IC_50_ values are calculated from sigmoidal fitting of growth curves. Data shown as mean ± S.E.M. of three independent experiments.

In the MTT assay, emodin exhibited a dose and time dependent decrease in cell proliferation as early as 24 h, continuing to 72 h of exposure, with IC_50_ values of 126, 61, and 18 μM with increasing time respectively for NCI-H-520 cells (Figure 4A), 97, 67, and 64 μM for NCI-H-460 cells (Figure 4B) and 124, 76, 54 μM at 24, 48 and 72 h respectively for the A-549 cells (Figure 4C). Interestingly, the NCI-H-460 cells were the most sensitive, whereas the A-549 were the least sensitive to emodin.

### Emodin Inhibited Cell Proliferation and Induced Cell Cycle Arrest

**Figure 5:**
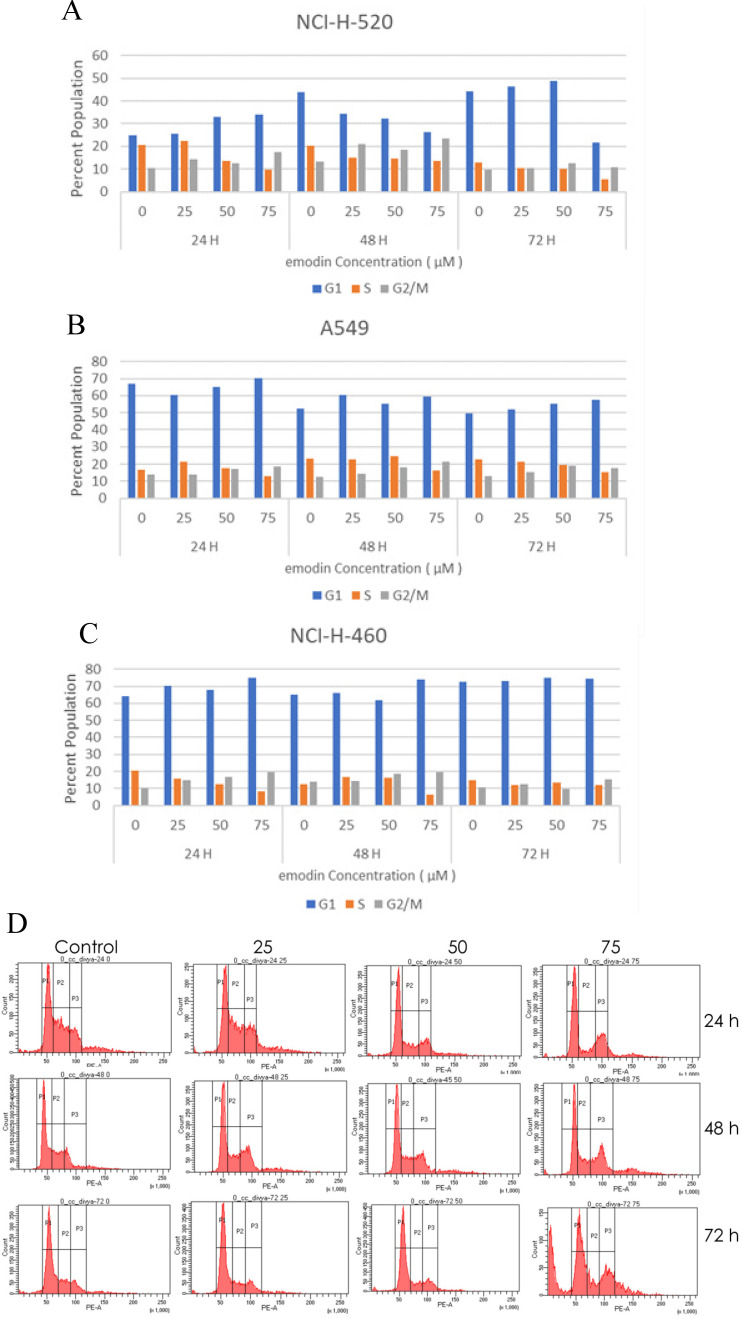

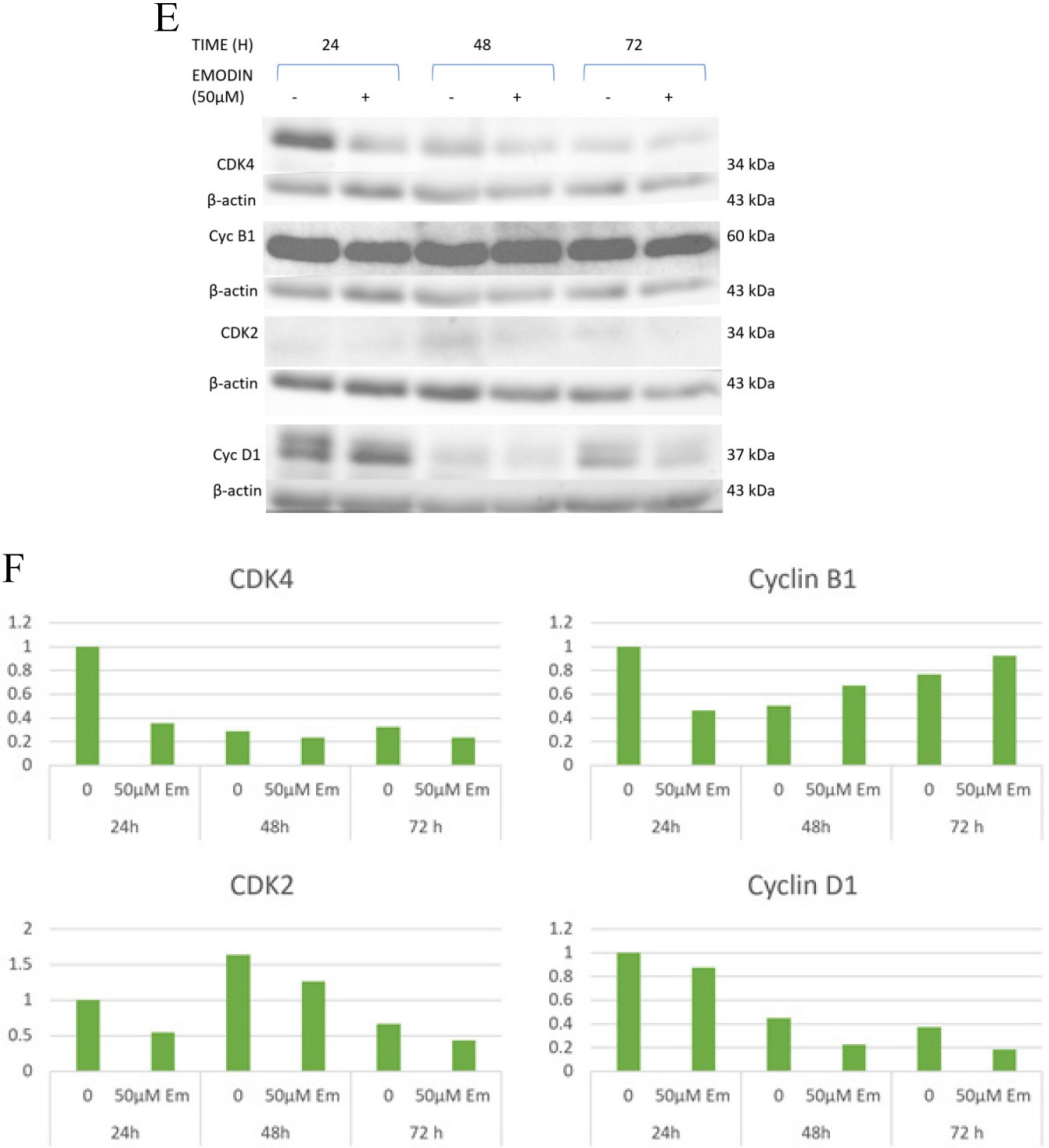
** Cell cycle analysis. A, B** and **C** are the graphical representation of the number of cells in G1, S and G2/M phases of NSCLC cells with emodin treatment (0, 25, 50 and 75 µM) at 24, 48 and 72 h, as measured by BD FACS Aria Flow Cytometer for NCI-H-520 (squamous cell carcinoma), A-549 (adenocarcinoma) and NCI-H-460 (Large cell carcinoma) cell lines respectively. **D.** Flow cytometric plots of NCI-H-520 cells with emodin treatment (0, 25, 50 and 75 µM) at 24, 48 and 72 h. Western Blot Analysis of Cell Cycle proteins. **E.** Protein level expression profile of CDK-4, Cyclin B1, CDK-2 and Cyclin D1 respectively. **F (a-d).** The graphical representation of the protein levels quantified by Image-J software. The data are representative of three independent experiments carried out under the same conditions.

The emodin treatment causes cell cycle arrest in NSCLC cells in a concentration and time dependent manner. Post-treatment with emodin the cycle of NCI-H-520 cells was altered. The percentage of cells in the G2/M phase in the emodin treated group was significantly higher than in the untreated group and the percentage of cells in the S phase emodin group was significantly lower than in the untreated vehicle control group. At 24, 48, and 72 h G2/M increased from approximately 19% to 28%, 17% to 37%, and 14% to 29%, respectively, and the S phase decreased from 37% to 16%, 26% to 21%, and 19% to 14%, respectively (Figure 5A and D).

In the A549 cells, the G2/M phase cells increased in proportion from 14% to 18%, 13% to 22%, and 15% to 20%, respectively, and the S phase cells gradually declined from 17% to 13%, 24% to 17%, and 27 to 17%, respectively, at 24, 48, and 72 h (Figure 5B). In NCI-H-460, the G2/M cells increased from 11% to 19%, 15% to 20%, and 11% to 15% at 24, 48, and 72 h respectively, and the proportion of the S phase cells decreased from 21% to 8%, 14% to 6%, and 15% to 12%, respectively (Figure 5C).

As binding emodin and MTH1 is demonstrated to induce cell cycle arrest at G2/M and decrease in the S phase in NSCLC cells, the expression and activation of cell cycle related proteins was investigated using Western blotting analysis (Figure 5E). While the protein level expression profile of cyclin-D1, CDK-2, and CDK-4 decreased in NCI-H-460 cells, cyclin-B1 levels increased on being treated with emodin in a duration dependent manner at 50 μM compared with untreated vehicle control cells (Figure 5F). This indicates that emodin could induce apoptosis in NSCLC cells in a duration and dose-dependent manner.

### Emodin Induced and Increased the Rate of Apoptosis in NSCLC Cells

Emodin-induced apoptosis in NSCLC cells was apparent by the characteristic features of apoptosis observed on the Hoechst 33342 staining. We observed cellular surface changes such as rounding, detachment, and membrane-blebbing as cells prepared to undergo apoptosis after treatment in a time and concentration dependent manner (Supplementary Figure 2A). Furthermore, post-nuclear staining with the Hoechst 33342 dye the number of stressed NSCLC cells increased from < 1 to > 33 in NCI-H-520, < 4 to > 41 in NCI-H-460, and < 3 to > 35 in the A-549 cell lines with increased treatment (Supplementary Figure 2B).

To verify whether emodin binding to MTH1 induced apoptosis of NSCLC cells, we did a primary experiment and stained cells with annexin V-FITC and PI, followed by fluorescent microscopy. The results are presented in Supplementary Figure 3A, where emodin induced a concentration dependent augmentation in the NCI-H-520 cell apoptosis in 24 h. There is an increasing green fluorescence (annexin positivity) and red fluorescence (PI positivity) with increasing emodin treatment. The mean fluorescent intensities are graphically represented in Supplementary Figure 3B, indicating an increase in the fluorescence signal of apoptotic cells with increasing emodin treatment for 24 h, as observed under the confocal microscope. The study concluded a significant apoptosis induction by emodin as observed by fluorescent microscopy in the NSCLC cells in a concentration dependent manner.

**Figure 6:**
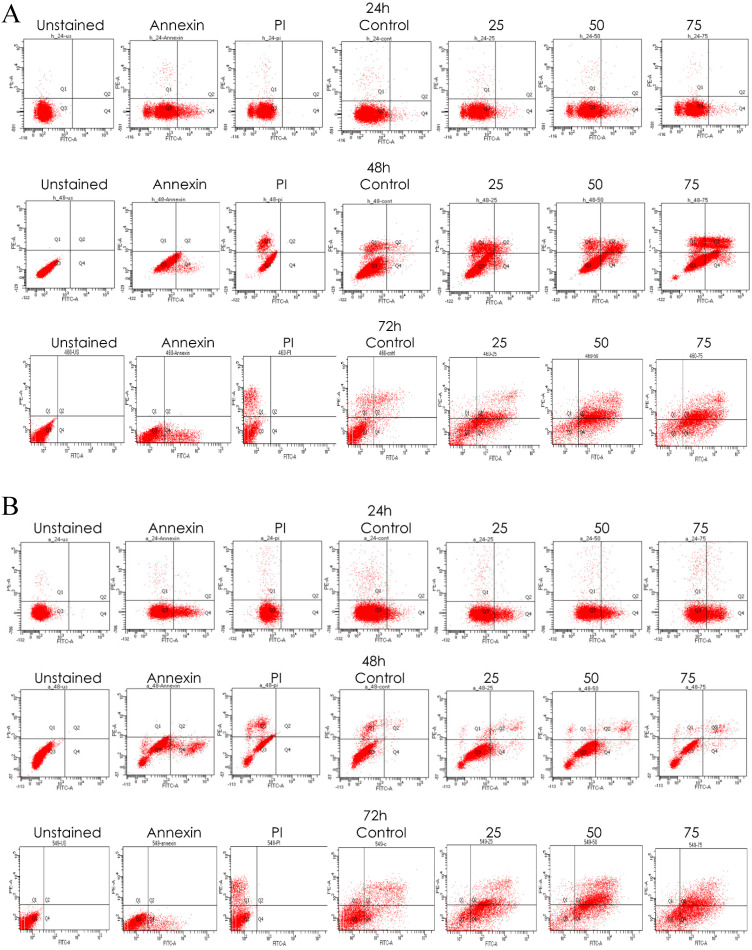

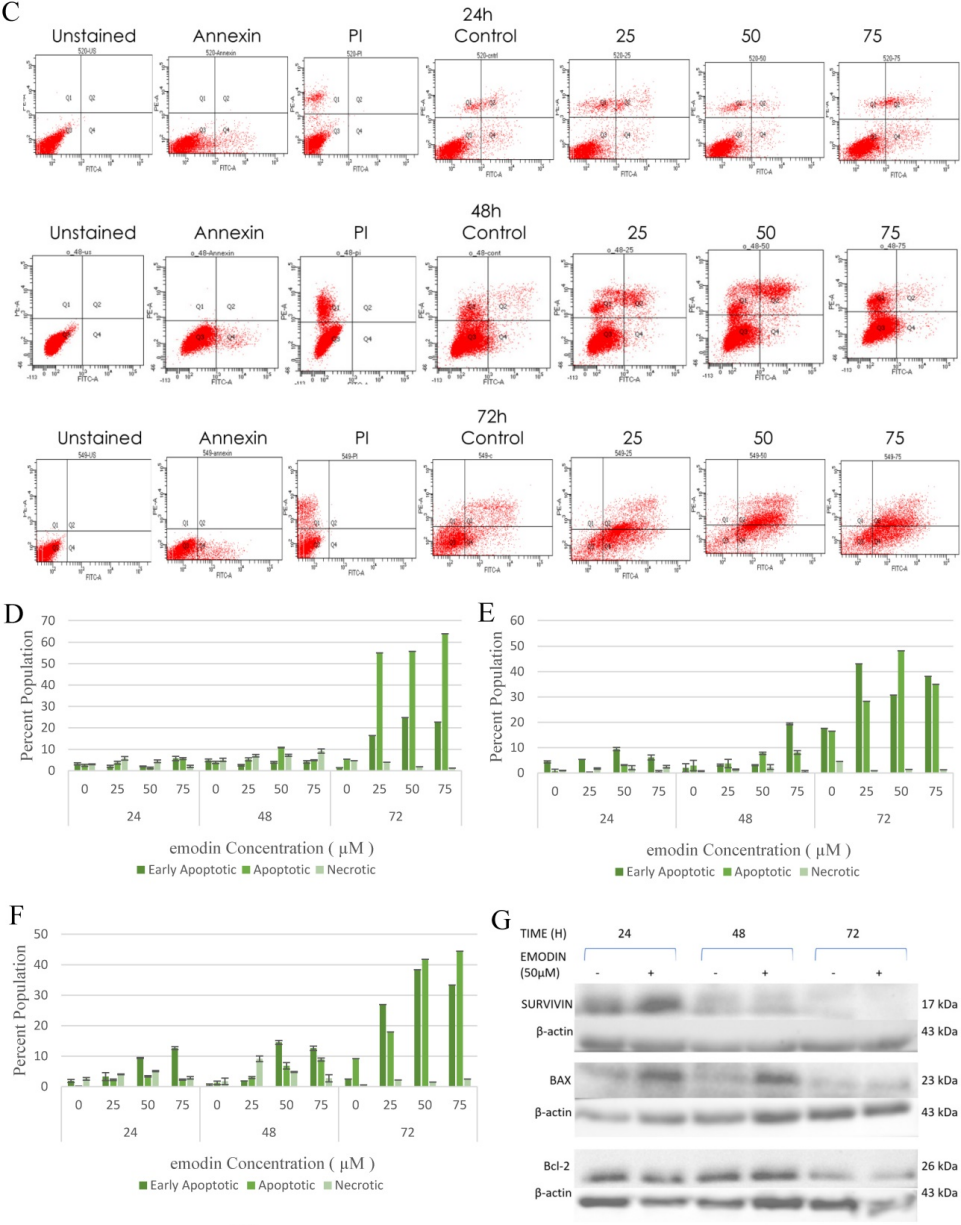

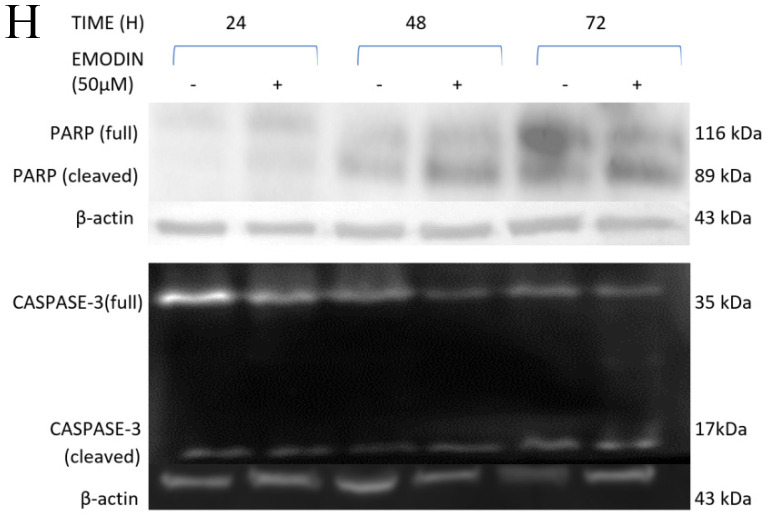

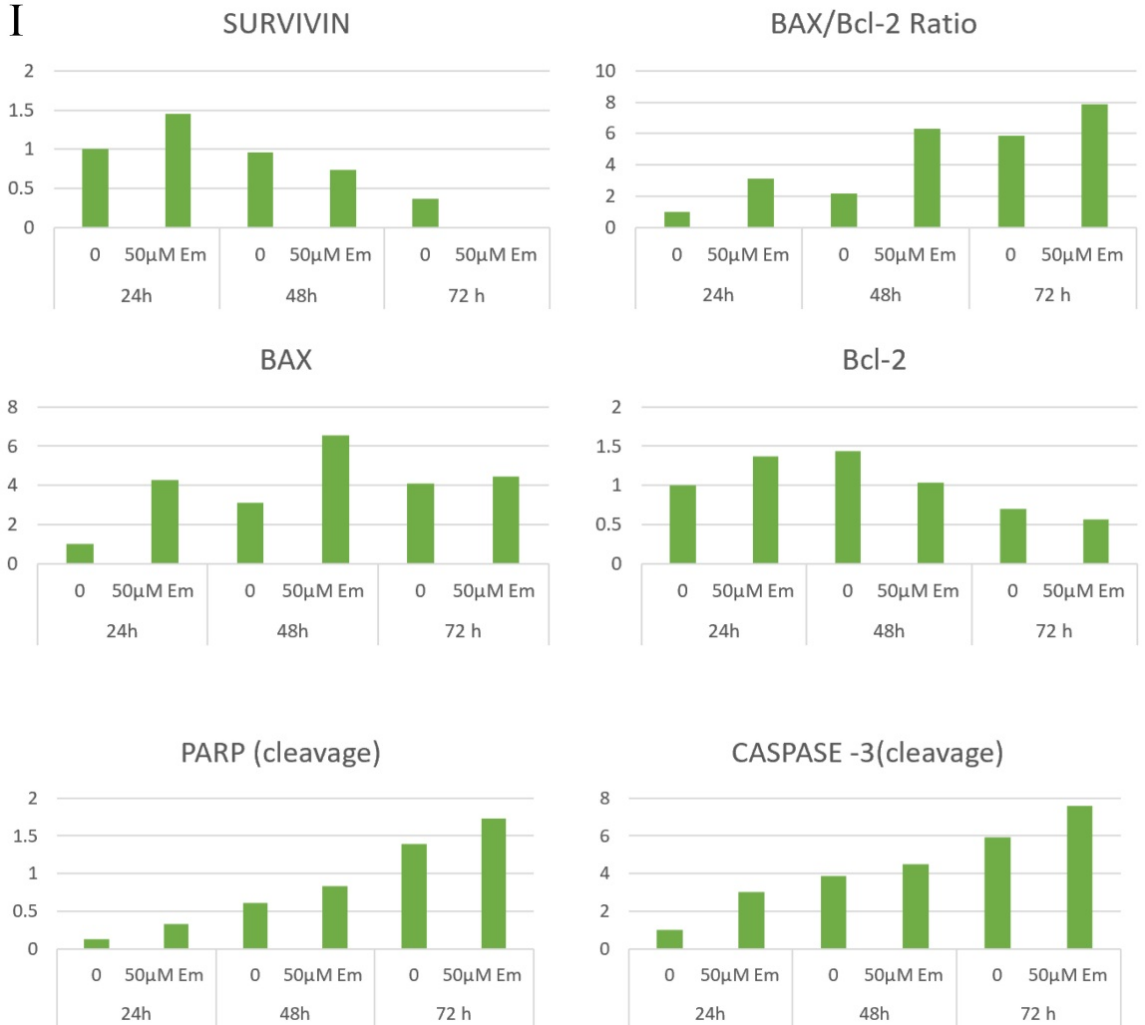
** Measurement of Apoptosis by Annexin V-PI assay. A**, **B** and **C** are Representative plots obtained from flow cytometry for NCI-H-460 (Large cell carcinoma), A-549 (adenocarcinoma) and NCI-H-520 (squamous cell carcinoma) cells under increasing emodin treatment (0, 25, 50 and 75 µM) at 24, 48 and 72 h respectively. The lower left quadrant indicates the viable cells. The lower right quadrant represents the annexin V positive/propidium iodide (PI) negative staining indicating early apoptotic cells. The upper right quadrant represents high annexin V and PI staining both, indicating late apoptosis and the upper left quadrant represents low annexin V and high PI staining indicating necrosis. **D**, **E** and **F** are the graphical representation of percent inhibition for NCI-H-520 (squamous cell carcinoma), A-549 (adenocarcinoma) and NCI-H-460 (Large cell carcinoma) cells respectively. **G and H.** Protein level expression profiles of Survivin, Bax, Bcl-2 and PARP and Caspase-3 cleavage respectively. **I:** The graphical representation of the protein levels quantified by Image-J software. Data shown as mean ± S.E.M. of three independent experiments.

We also performed a confirmatory flow cytometric analysis. As highlighted on the graphs in Figure 6A-C, only a very small number of apoptotic cells were detected in the vehicle control group. Based on distinct double-staining patterns in NCI-H-520 treated with emodin, there was a dose and duration dependent increase in the number of apoptotic cells, with their population increasing from ~2.5% to ~5.6%, ~3.8% to ~2.9%, and ~5.4% to ~63.9% at 24, 48, and 72 h, respectively (Figure 6D). In the A-549 cells the increase in the number of apoptotic cells went from ~0.8% to ~3.1%, ~2.9% to ~8.1%, and ~16.5% to ~35.0%, respectively (Figure 6E), and in NCI-H-460 the increase was from ~0.1% to ~2.3%, ~1.2% to ~8.9%, and ~9.2% to ~44.5% at 24, 48, and 72 h respectively (Figure 6F). These results indicate emodin-induced apoptosis in NSCLC cells.

As emodin has been demonstrated to induce apoptosis in NSCLC cells, the expression and activation of apoptosis related proteins was investigated using Western blotting analysis. The protein level expression profile of Bax (pro-apoptotic protein) and survivin increased and Bcl-2 (anti-apoptotic protein) decreased in the NCI-H-520 cells treated with emodin in a duration dependent manner at 50 μM, compared with the untreated vehicle control cells. The cleavage of caspase-3 and PARP (poly ADP ribose polymerase) revealed that cleaved-caspase-3 and cleaved-PARP levels increased in emodin treated NCI-H-520 cells compared with the untreated vehicle control cells (Figure 6G-I). We also observed an increase in the P-21 protein levels while p-65 NFκβ protein levels decreased with time on emodin treatment (Supplementary Figure 4A). These results suggest that emodin may activate the caspase-dependent apoptosis pathway in NSCLC cells.

### Emodin Inhibited the Migratory Activity of NSCLC Cells

**Figure 7:**
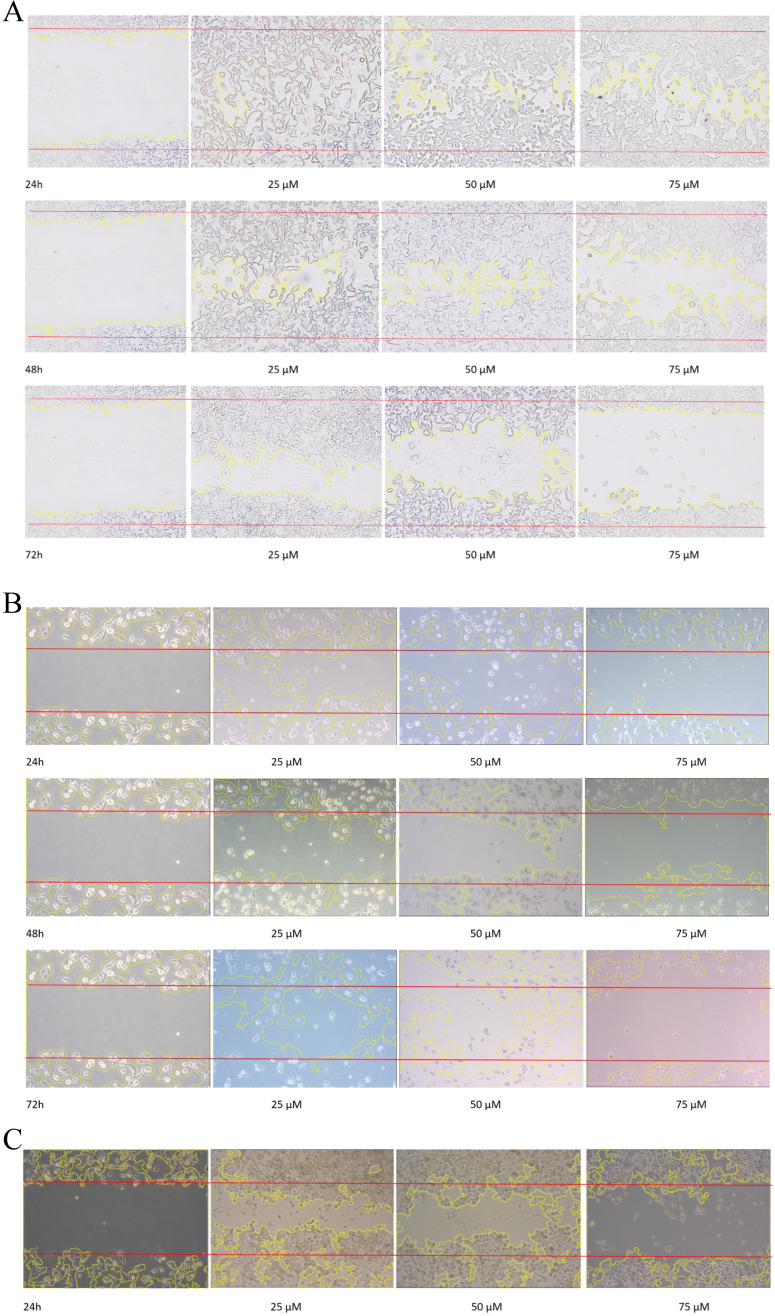

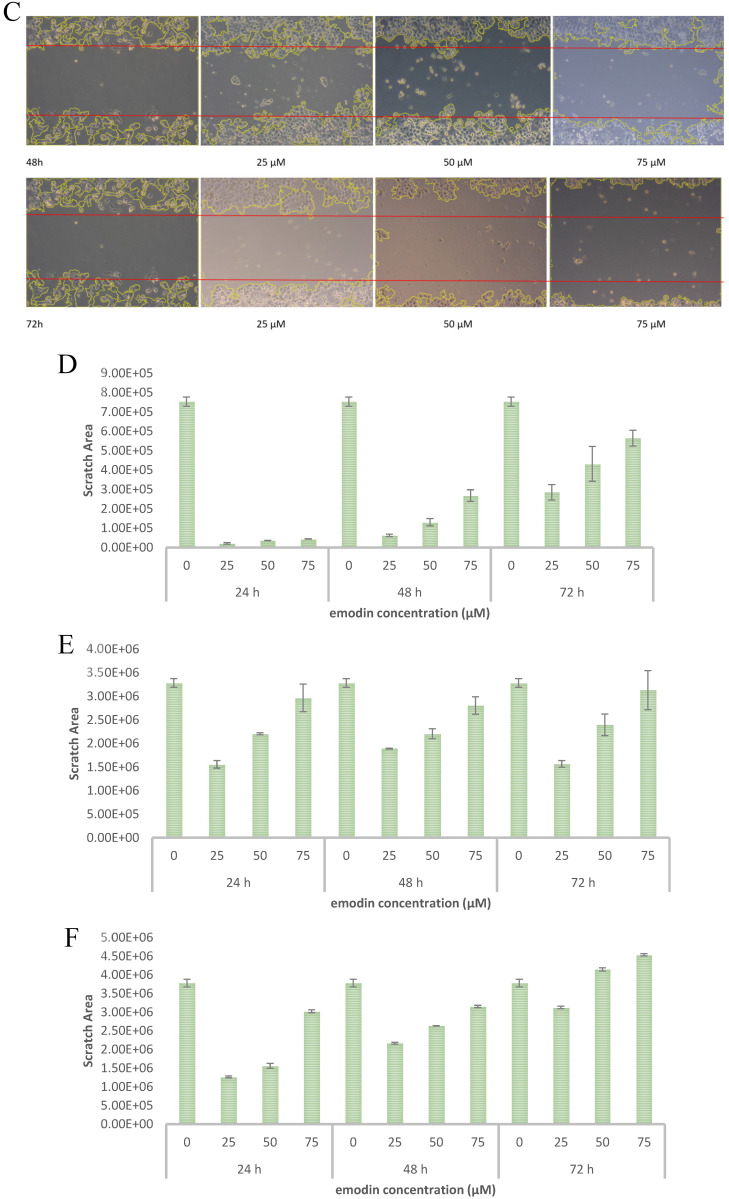

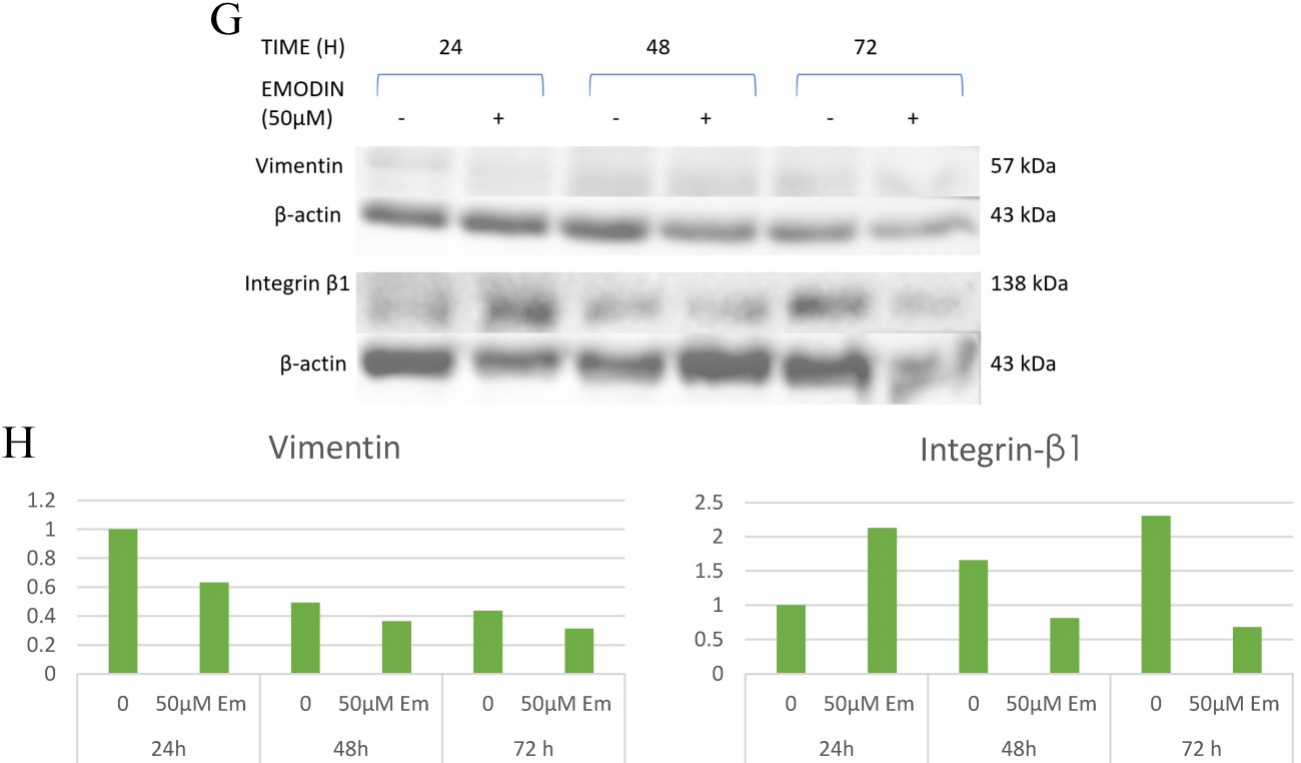
** Wound scratch assay. A**, **B** and **C** are photographical representation of the visual wound healing under increasing emodin treatment (0, 25, 50 and 75 µM) at 24, 48 and 72 h, as measured by phase-contrast for NCI-H-520 (squamous cell carcinoma), A-549 (adenocarcinoma) and NCI-H-460 (Large cell carcinoma) cell lines respectively. **D, E** and **F** are the graphical representation of scratch areas for A, B and C respectively. **G.** Protein level expression profiles of Vimentin and Integrin β1 respectively. **H.** The graphical representation of the protein levels quantified by Image-J software. Data shown as mean ± S.E.M. of three independent experiments.

To evaluate the effect of emodin on cell migration, a wound healing assay was performed on the NSCLC cell lines. The wound healing assay suggested that emodin suppresses the migration of NSCLC cells in a dose and duration dependent manner as compared with their corresponding untreated controls (Figure 7A-C). Treatment with 50 µM emodin visually inhibited NCI-H-520 cell migration, with wound areas of ~5% in 24, ~17% in 48, and ~57% at 72 h. The effect of emodin inhibition was ~57% in 24, ~67% in 48, and ~73% at 72 h on A-549 scratch areas. In NCI-H-460, the cell migration was significantly inhibited with the wound area ~41% at 24 h, ~70% at 48 h, and ~110% at 72 h (Figure 7D-F).

Given the effects of emodin on NSCLC cell migration, the cell migratory mechanism was further investigated by Western blot assay. Since integrins and vimentin play an important role in cancer cell metastasis, the protein expression levels were detected by Western blotting. In the NCI-H-520 cells, emodin duration dependently reduced the integrin β1 and vimentin protein expression levels at a concentration of 50 µM in treated cells compared with the untreated vehicle control (Figure 7G). These data therefore suggest that emodin had an inhibitory effect on the migration of NSCLC, which was associated with the downregulation of integrin β1 and vimentin protein expression.

### The Effects of Emodin on NSCLC Cells Produced Though Activation of ROS

**Figure 8:**
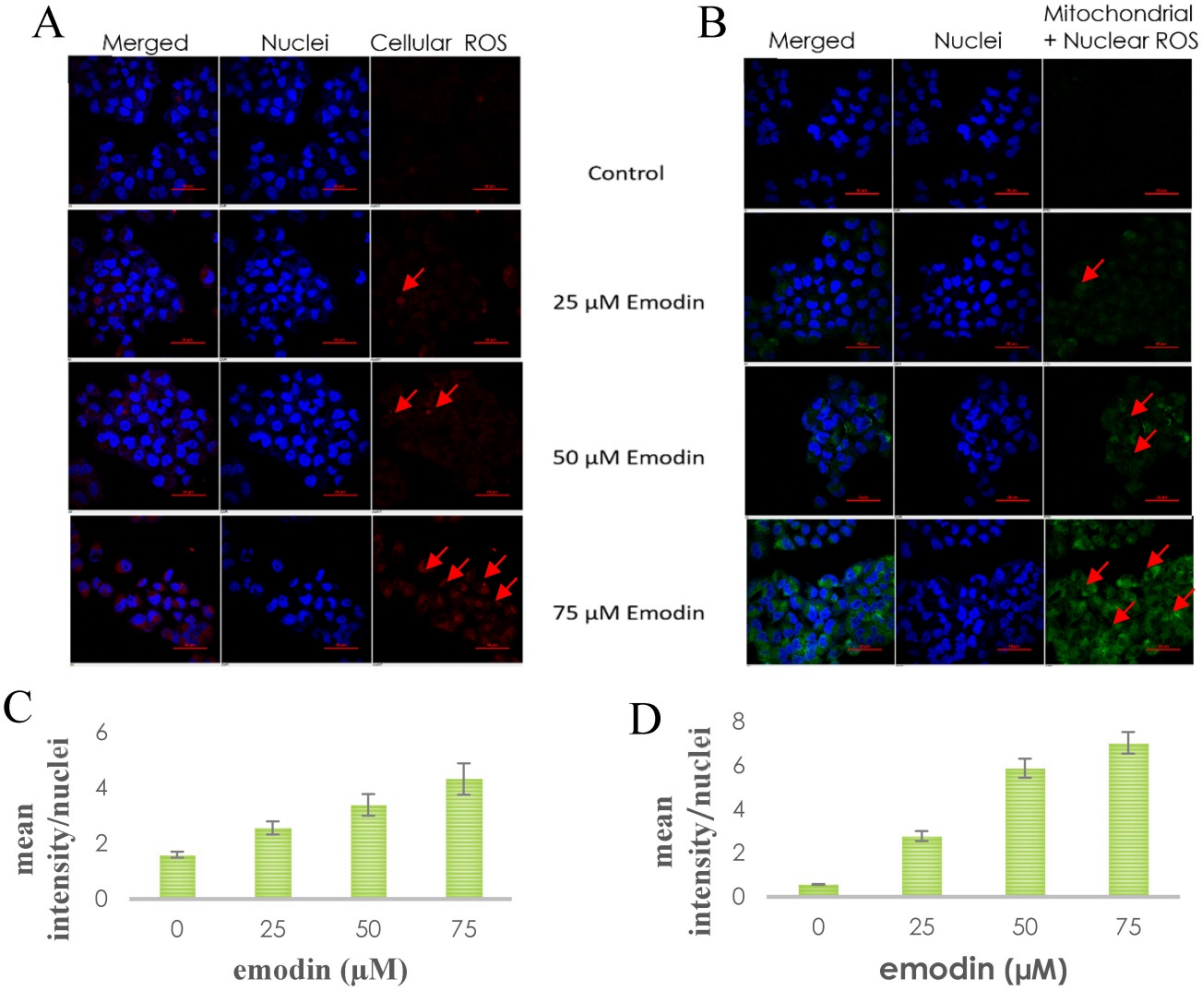
**A.** Confocal microscopic images of NCI-H-520 cell line stained with CellROX™ Deep Red, showing increasing red fluorescence (increasing ROS). **B.** Confocal microscopic images of NCI-H-520 cell line stained with CellROX™ Green, showing increasing green fluorescence (increasing ROS). **C** and **D.** Graphical representation of the mean fluorescent intensity/ nuclei indicating an increase in the fluorescence signal of ROS in cells with increasing emodin treatment for 24 h, as observed under the confocal microscope for A and B respectively.

To verify whether emodin-MTH1 binding caused overproduction of ROS, a primary investigation was carried out on the amount of intracellular and mitochondrial ROS levels generated on emodin treatment. The results are presented in Figure 8A-B, where emodin induced a concentration dependent augmentation in NCI-H-520 ROS in 24 h. There is an increasing red fluorescence (cellular ROS positivity) and green fluorescence (mitochondrial and nuclear ROS positivity) with increasing emodin treatment. The mean fluorescent intensities were graphically represented in Figure 8C-D, with mean fluorescent intensity/nuclei indicating an increase in the fluorescence signal of ROS in cells with increasing emodin treatment for 24 h, as observed under the confocal microscope.

### Emodin Caused Depletion in the Mitochondrial Membrane Potential (ΔΨm) of NSCLC Cells

**Figure 9:**
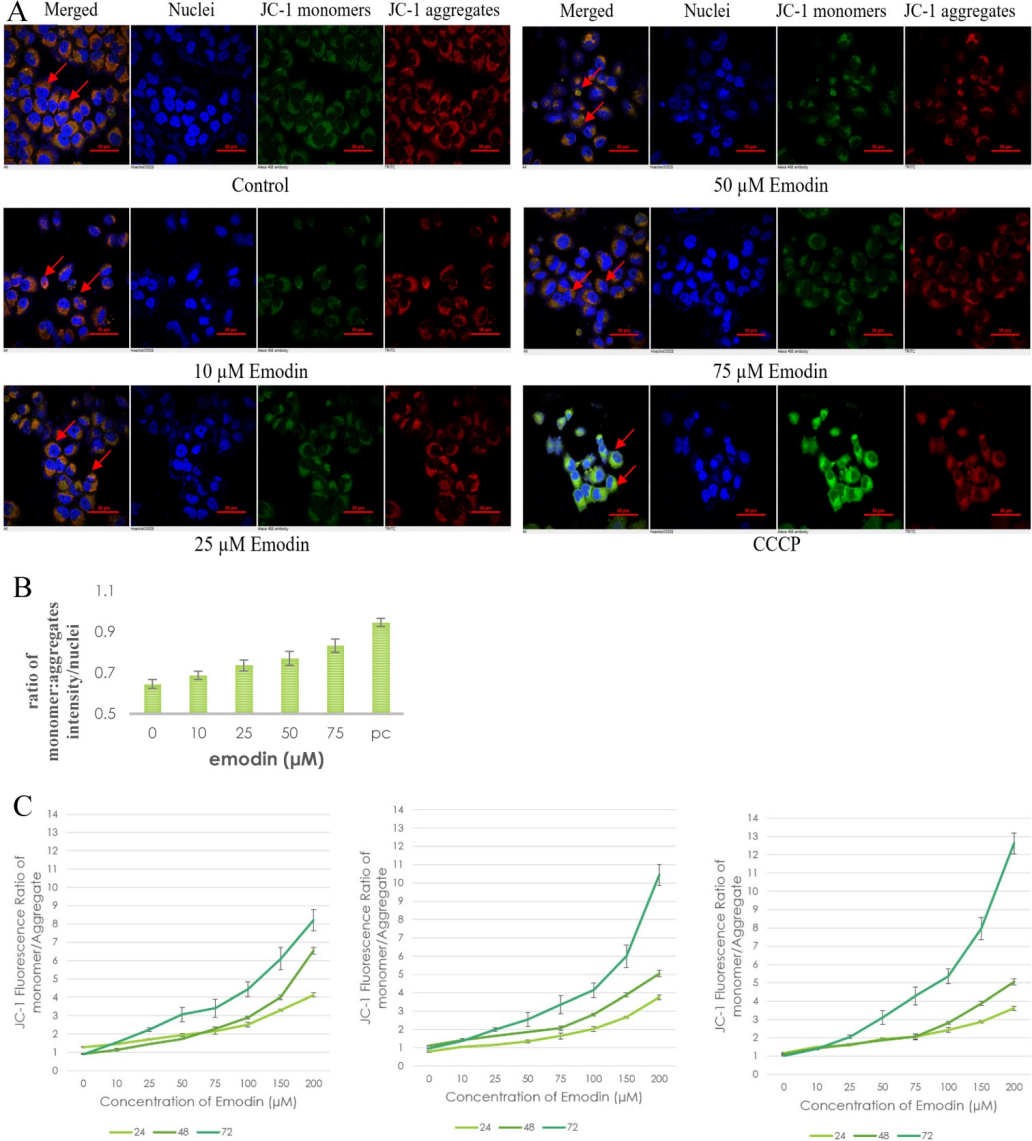
** Mitochondrial membrane potential analysis by JC-1 staining. A.** Confocal microscopy images of NCI-H-520 (squamous cell carcinoma) cells with emodin treatment (0, 10, 25, 50 and 75 µM) and CCCP (positive control) at 24h, stained with JC-1 dye. **B.** graphical representation of the fluorescent intensities depicted as the ratio of monomer:aggregates from the Confocal microscopy images of NCI-H-520 (squamous cell carcinoma) cells represented in A. **C**, **D** and **E** shows the graphical representation plots of fluorescent intensities (ratio of monomer:aggregates) obtained from fluorimetry for NCI-H-520 (squamous cell carcinoma), A-549 (adenocarcinoma) and NCI-H-460 (large cell carcinoma) cells under increasing emodin treatment (0, 10, 25, 50, 75, 100, 150 and 200 µM) at 24, 48 and 72 h respectively. The data shown as mean ± S.E.M. of three independent experiments carried out under the same conditions.

To investigate the possible role of emodin generated ROS in DNA damage and subsequent apoptosis in NSCLC cells, preliminary fluorescent microscopy and fluorimetry using JC-1 dye was performed. The NCI-H-520 cells were treated with emodin and carbonyl cyanide 3-chlorophenylhydrazone (CCCP) at 24 h and stained with JC-1 dye. We observed a concentration dependent increase in the fluorescent intensity ratio of monomer aggregates in comparison to the vehicle control. The highest fluorescence ratio was observed in cells treated with protonophore CCCP acting as the positive control (Figure 9A). There was an increase in the fluorescent intensity ratio of monomer:aggregates from ~0.64 in the vehicle control to ~0.83 in 75 μM emodin treated cells and ~0.94 in the CCCP control (Figure 9B).

The NSCLC cells were used for fluorometric analysis using JC-1 staining to study the effect of emodin on mitochondrial membrane potential (∆*ψ_m_*). A dose and duration dependent increase in the fluorescent intensity ratio of JC-1 monomer:aggregates were obtained from fluorimetry. The result indicates that the mitochondrial membrane potential (∆*ψ_m_*) was significantly reduced as measured by the increased fluorescence ratios of the JC-1 dye (Figure 9C-E).

**Figure 10:**
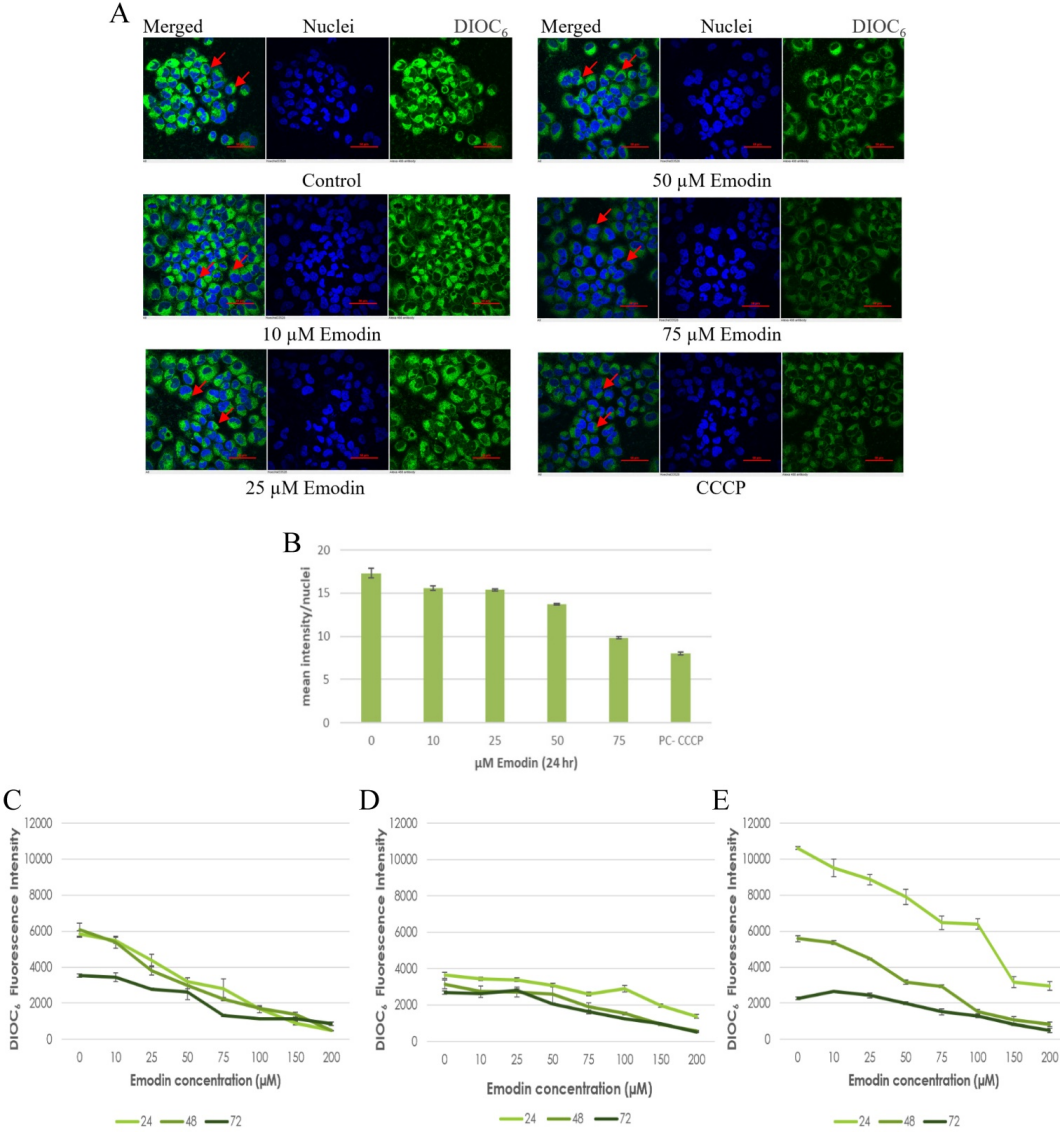
** Mitochondrial membrane potential analysis by DIOC_6_ staining. A.** Confocal microscopy images of NCI-H-520 (squamous cell carcinoma) cells with emodin treatment (0, 10, 25, 50 and 75 µM) and CCCP (positive control) at 24h, stained with DIOC_6_ dye. **B.** Graphical representation of the fluorescent intensities of the Confocal microscopic images of NCI-H-520 (squamous cell carcinoma) cells represented in **A. C, D and E** shows the graphical representation plots of fluorescent intensities obtained from fluorimetry for NCI-H-520 (squamous cell carcinoma), A-549 (adenocarcinoma) and NCI-H-460 (Large cell carcinoma) cells under increasing emodin treatment (0, 10, 25, 50, 75, 100, 150 and 200 µM) at 24, 48 and 72 h respectively. The data shown as mean ± S.E.M. of three independent experiments carried out under the same conditions.

To confirm the results of the JC-1 dye, preliminary fluorescent microscopy and fluorimetry using the DIOC_6_ dye were also performed. The NCI-H-520 (squamous cell carcinoma) cells were treated with emodin and CCCP (positive control) and we observed a concentration dependent decrease in the fluorescent intensities in comparison to the vehicle control. The lowest fluorescence was observed in cells treated with protonophore CCCP acting as the positive control (Figure 10A). There was a decrease in the fluorescent intensities from ~17 in the vehicle control to ~10 in 75 μM emodin treated cells and ~7 in the CCCP control (Figure 10B).

Furthermore, the NSCLC cells were used for fluorometric analysis using DIOC_6_ staining to study the effect of emodin on the mitochondrial membrane potential (∆*ψ_m_*). We observed a dose and duration dependent decline in the fluorescent intensities and the result indicates that the mitochondrial membrane potential (∆*ψ_m_*) was significantly reduced as measured by the reduced fluorescence of the DIOC_6_ dye (Figure 10C-E).

The drop in ∆ψm followed a dose and duration dependent pattern and decreased gradually, confirming that the cellular damage occurring at the mitochondrial level may be co-related to the apoptotic response of NSCLC cells to emodin. These data support the hypothesis that ROS generation by emodin causes mitochondrial membrane depolarization and the subsequent induction of apoptosis in NSCLC cells. It is thus concluded that emodin treatment led to a dose and duration dependent loss of ∆* ψ_m_*.

### The Effects of Emodin on NSCLC Cells Produced Though ROS Mediated Accumulation of Damaged DNA

**Figure 11:**
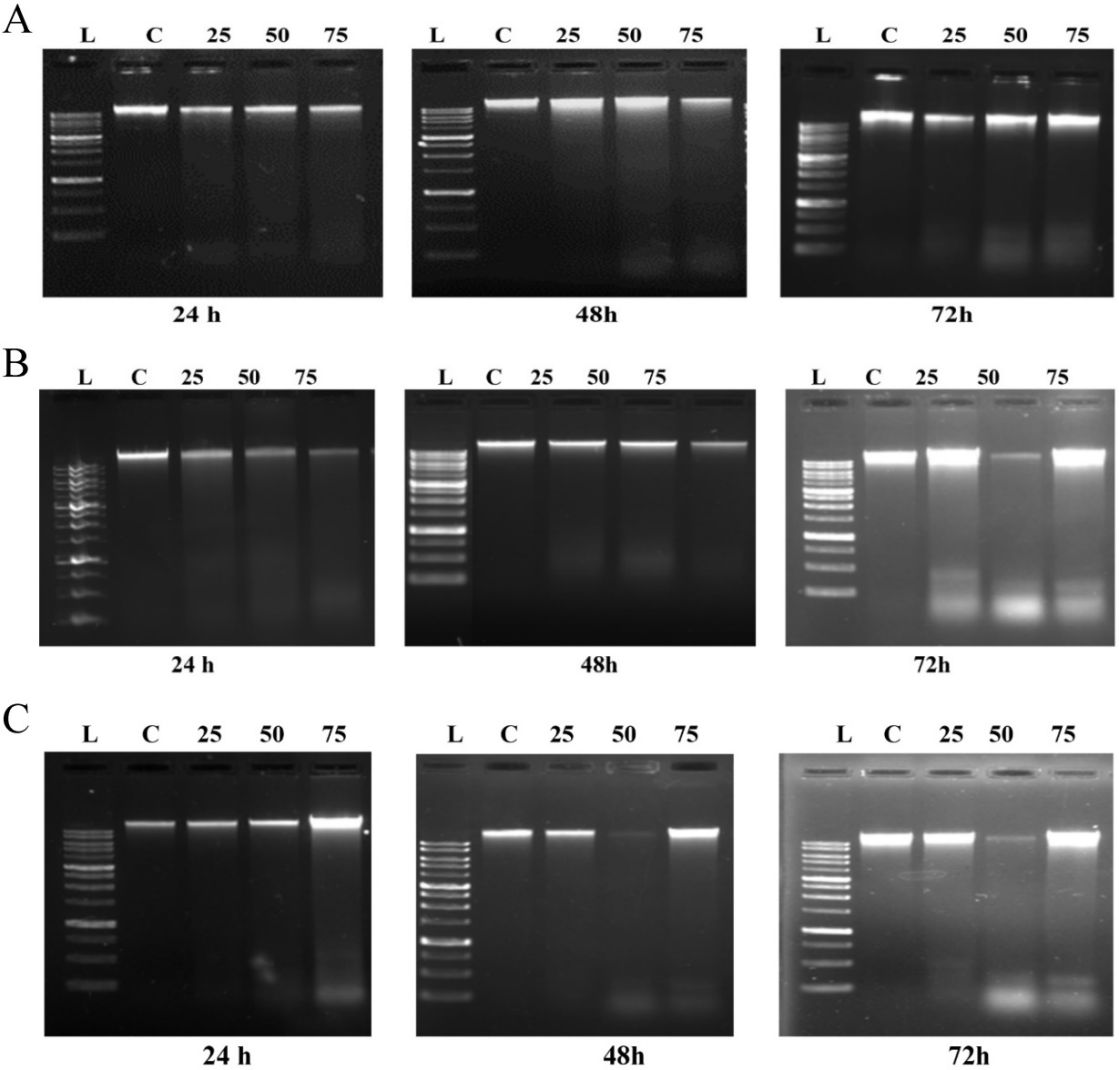
** Analysis of DNA fragmentation induced by emodin treatment. A, B and C** are photographical representation of the visual ladder like DNA bands formed under increasing emodin treatment (0, 25, 50 and 75 µM) at 24, 48 and 72 h, as assessed by 1% agarose gel electrophoresis and ethidium bromide staining for NCI-H-520, A-549 and NCI-H-460 cell lines respectively. L: Ladder, C: Control, 25: 25 µM emodin, 50: 50 µM emodin, 75: 75 µM emodin. The data are representative of three independent experiments carried out under the same conditions.

To further investigate the extent of ROS mediated induction of cell death caused by emodin, a DNA fragmentation assay was performed using agarose gel electrophoresis. As shown in Figure 11A, in NCI-H-520 (squamous cell carcinoma) emodin treatment at 24 h did not generate any DNA fragmentation, but at 48 and 72 h it generated a marked pattern of DNA fragmentation with a dose dependence, while the vehicle control alone was unable to induce any DNA degradation. In A-549 (adenocarcinoma) treatment with increasing emodin concentrations at the 24, 48, and 72 h time points clearly caused DNA fragmentation, which was similar to the ladder-like pattern observed in NCI-H-460 (large cell carcinoma) (Figure 11B-C).

**Figure 12:**
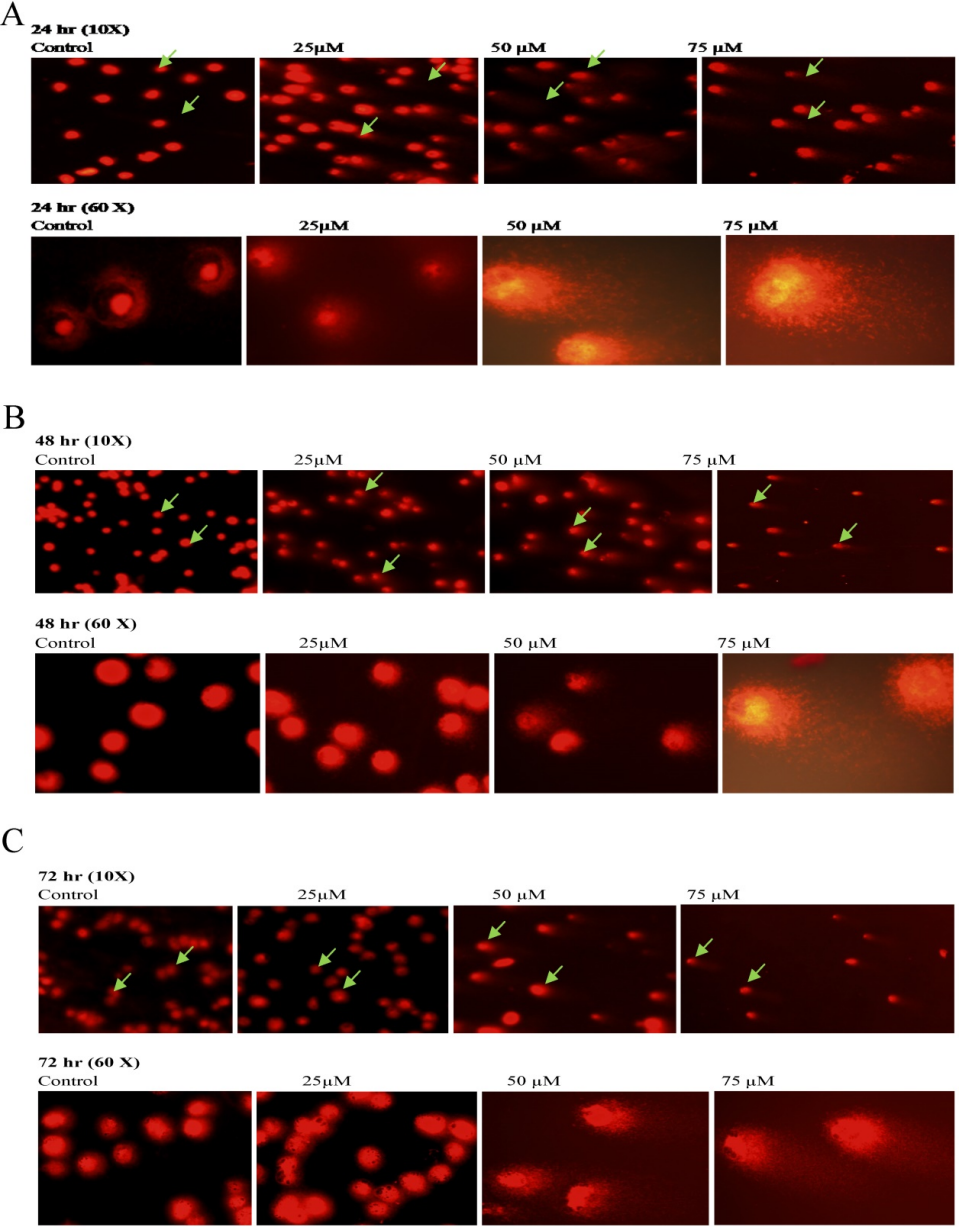

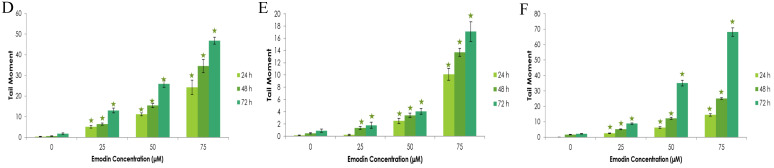
** Analysis of Comet Assay. A, B and C** are photographical representation of the visual comet like DNA from cells formed under increasing emodin treatment (0, 25, 50 and 75 µM) at 24, 48 and 72 h (at 10X and 60X), as assessed by single cell gel electrophoresis and ethidium bromide staining, observed under confocal microscope for NCI-H-520 (squamous cell carcinoma). **D**, **E** and **F** are graphical representations of the comet tail moments of NCI-H-460 (Large cell carcinoma), A-549 (adenocarcinoma) and NCI-H-520 (squamous cell carcinoma) cell lines respectively. Data shown as mean ± S.E.M. of three independent experiments and * indicate P values < 0.05.

To further confirm the potentiating effects of emodin on DNA damage in NSCLC cells, a single cell acrylamide gel electrophoresis (SAGE) assay was performed to be quantitatively and qualitatively analyzed. The emodin treated cells showed well-formed comets, with tail lengths increasing in a concentration and duration dependent manner in comparison to the untreated vehicle controls with no comet like appearance (Figure 12A-C). Post treatment, including the comet tail moments, that is tail lengths of NCI-H-460 in 24, 48, and 72 h, were increased from approximately 0.1 to 14.4, 1.6 to 25.0, and 2.1 to 68.1, respectively (Figure 12D). In A549 cells the comet tail moments increased from 0.3 to 24.1, 0.5 to 34.5, and 1.8 to 46.8, respectively (Figure 12E) and in NCI-H-520 cells, the comet tail moments increased from 0.1 to 10.1, 0.4 to 13.7, and 0.9 to 17 in 24, 48, and 72 h, respectively (Figure 12F). These observations exhibit emodin-induced NSCLC cell death, which could be mediated through an apoptotic pathway causing extensive DNA fragmentation and damage.

**Figure 13:**
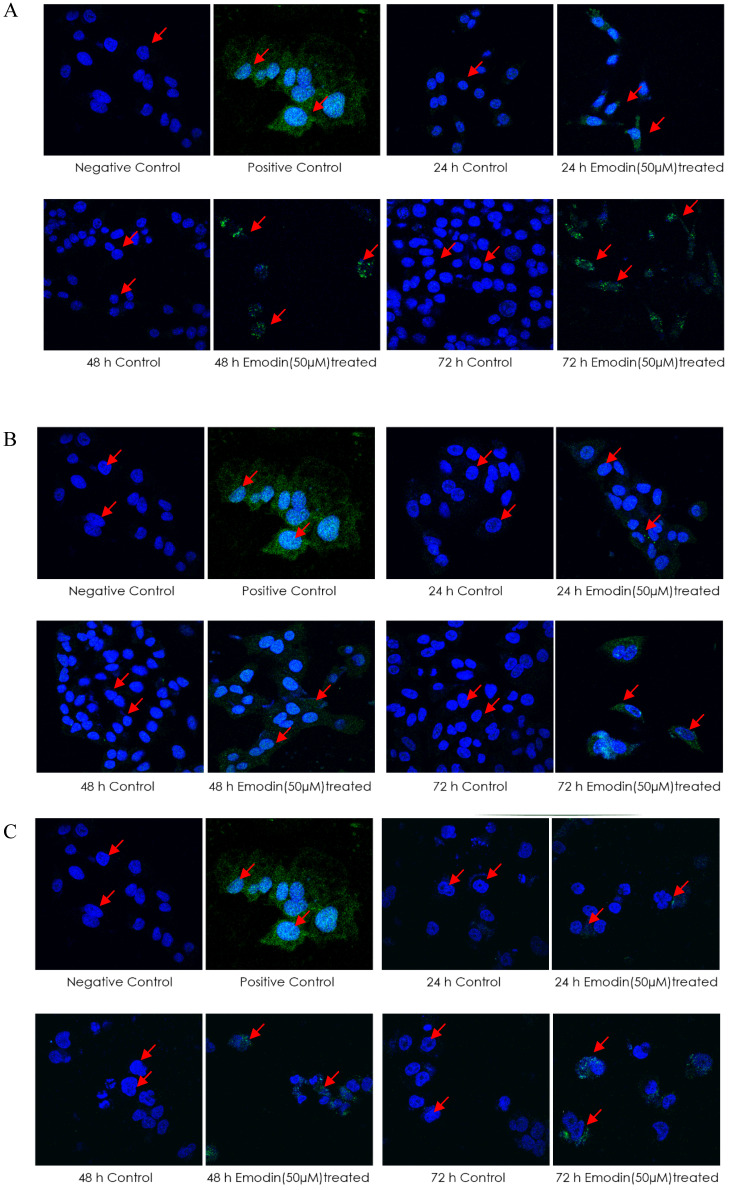

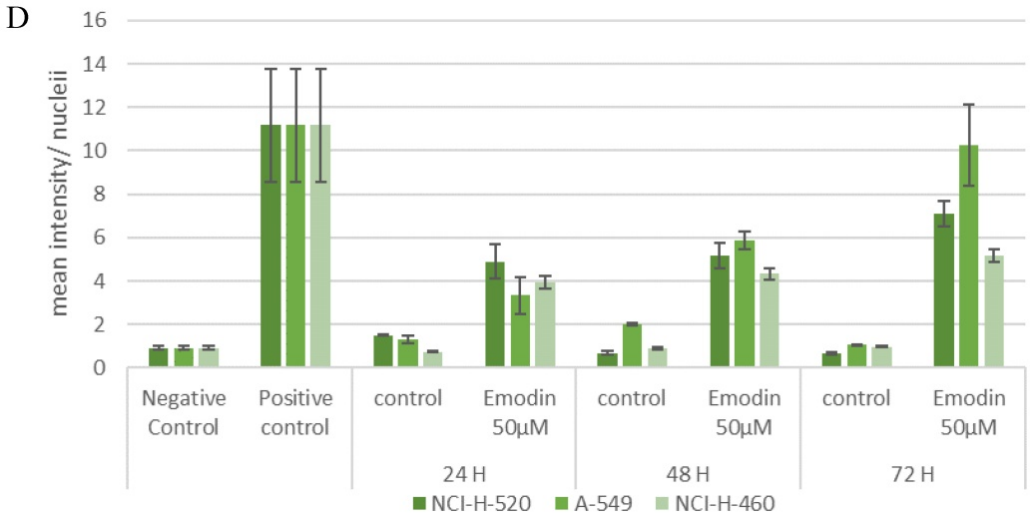
** Analysis of TUNEL Assay. A, B and C** are the Confocal microscopy images of NCI-H-520 (squamous cell carcinoma), A-549 (adenocarcinoma) and NCI-H-460 (Large cell carcinoma) cells under emodin treatment (50 µM) at 24, 48 and 72 h along with vehicle controls respectively stained with the TUNEL assay protocol. Increasing Green fluorescence indicates increasing DNA Damage. **D.** graphical representation plot of fluorescent intensities obtained from confocal microscopy images for A-C. The data shown as mean ± S.E.M. of three independent experiments carried out under the same conditions.

A TUNEL (terminal deoxynucleotidyl transferase dUTP nick end labeling) assay was then performed for the detection of DNA strand breaks induced by emodin treatment. The cells treated with emodin showed a distinct appearance in nuclear condensation and incorporation of the Alexa-Fluor labeled nucleotides into the DNA (a positive TUNEL reaction) in a duration dependent manner compared to the vehicle control (Figure 13A-C). Post-treatment with emodin, the mean intensity/nuclei of TUNEL reaction positive cells for the NCI-H-520 cells in 24, 48, and 72 h were increased from approximately 1.5 to 4.9, 0.6 to 5.1, and 0.6 to 7.0, respectively. In the A549 cells, the mean intensity/nuclei of TUNEL reaction positive cells increased evidently with values from 1.3 to 3.3, 2.0 to 5.8, and 1.0 to 10.2 and in the NCI-H-460 cells, the mean intensity/nuclei increased from 0.7 to 3.9, 0.9 to 4.3, and 0.9 to 5.1 in 24, 48, and 72 h, respectively (Figure 13D). These observations exhibit that emodin binding to MTH1 increases ROS that eventually induces nuclear DNA fragmentation, a hallmark of apoptosis in NSCLC cells. Thus, NSCLC cell death is possibly mediated through an apoptotic pathway causing extensive DNA fragmentation and damage.

Since emodin is known to produce ROS, the relationship between ROS generation and DNA damage and repair eventually leading to apoptosis requires further understanding. Initial experiments indicated that emodin induces ROS and subsequent DNA damage that activates DNA double stranded break repair mechanisms, which fail to repair the DNA owing to extensive damage caused by emodin mediated MTH1 functional inhibition; thus, leading to apoptosis in NSCLC cells.

**Figure 14:**
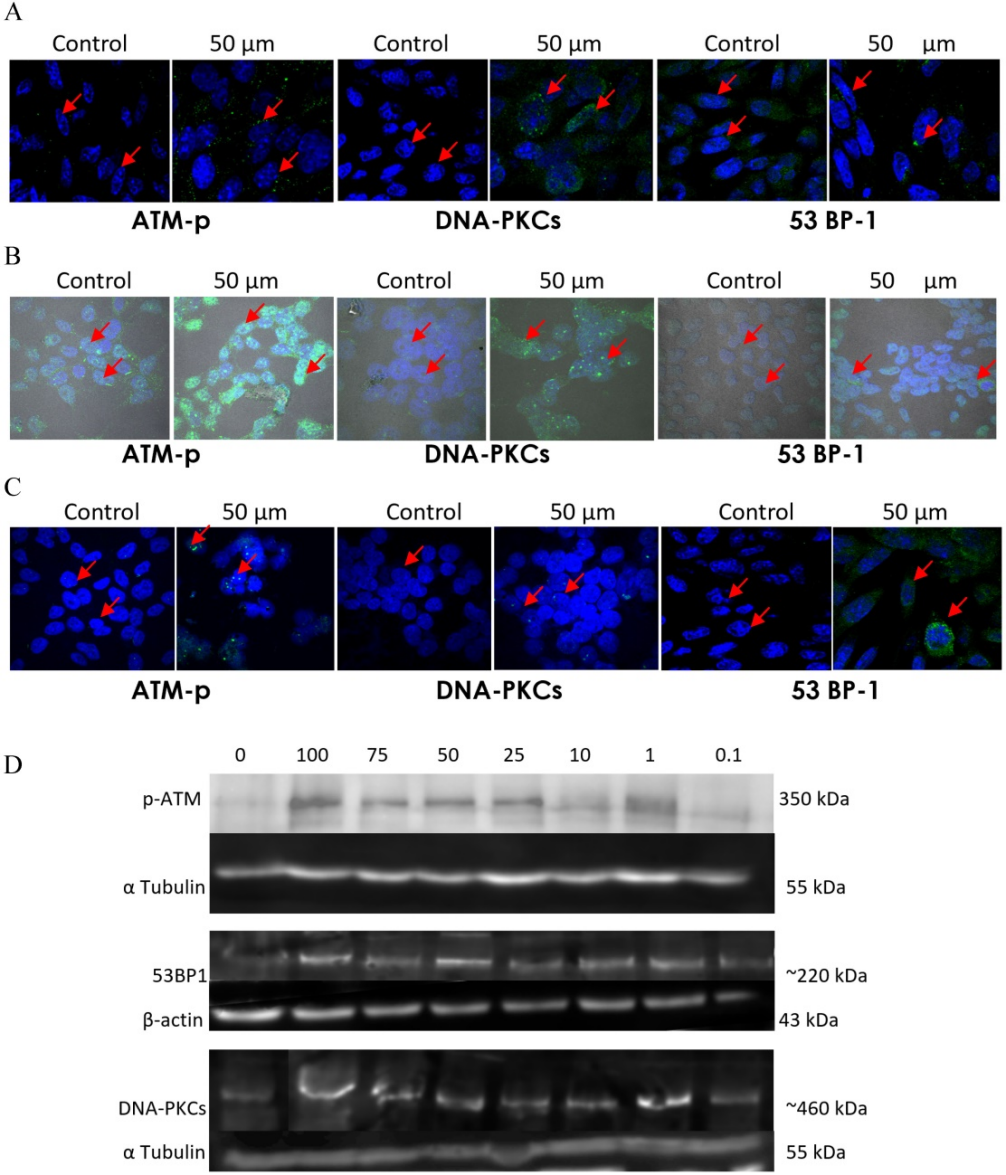
** Analysis of DNA damage and repair activation. A, B and C** are the Confocal microscopy images of NCI-H-520 (squamous cell carcinoma), A-549 (adenocarcinoma) and NCI-H-460 (Large cell carcinoma) cells under emodin treatment (50 µM) at 24 h along with vehicle controls respectively stained with ATM-p, DNA-PKCs and 53 BP-1 Primary antibodies subsequently stained with Alexa-488 labelled Secondary antibodies. Increasing Green fluorescence indicates increasing expression of the DNA Damage and repair induction specific proteins. **D.** Protein level expression profiles of ATM-p, DNA-PKCs and 53 BP-1 respectively. The data shown is representative of three independent experiments carried out under the same conditions.

To validate this further, the expression and activation of DNA damage and repair related proteins was investigated by immunofluorescence labelling followed by confocal microscopy and Western blotting analysis (Figure 14). Immunofluorescence study of the DNA damage and repair proteins ATM-p, DNA-PKCs, and 53 BP-1 revealed an increase in NSCLC cells treated with emodin in a duration dependent manner at 50 μM, compared with untreated vehicle control cells (Figure 14A-C).

In addition, to the given the effects of emodin on DNA damage and repair proteins in NSCLC studied by immunofluorescence, protein levels were further investigated by western blot. The western blotting analysis of the ATM-p, DNA-PKCs and 53 BP-1 protein expression profiles revealed similar results. The DNA damage and repair protein expression levels increased in Emodin treated NSCLC cells compared with untreated vehicle controls (Figure 14D). These results suggested that Emodin may activate the double stranded DNA damage and repair pathway in NSCLC cells.

### Expression and Purification of Human MTH1 Protein from E. Coli

**Figure 15:**
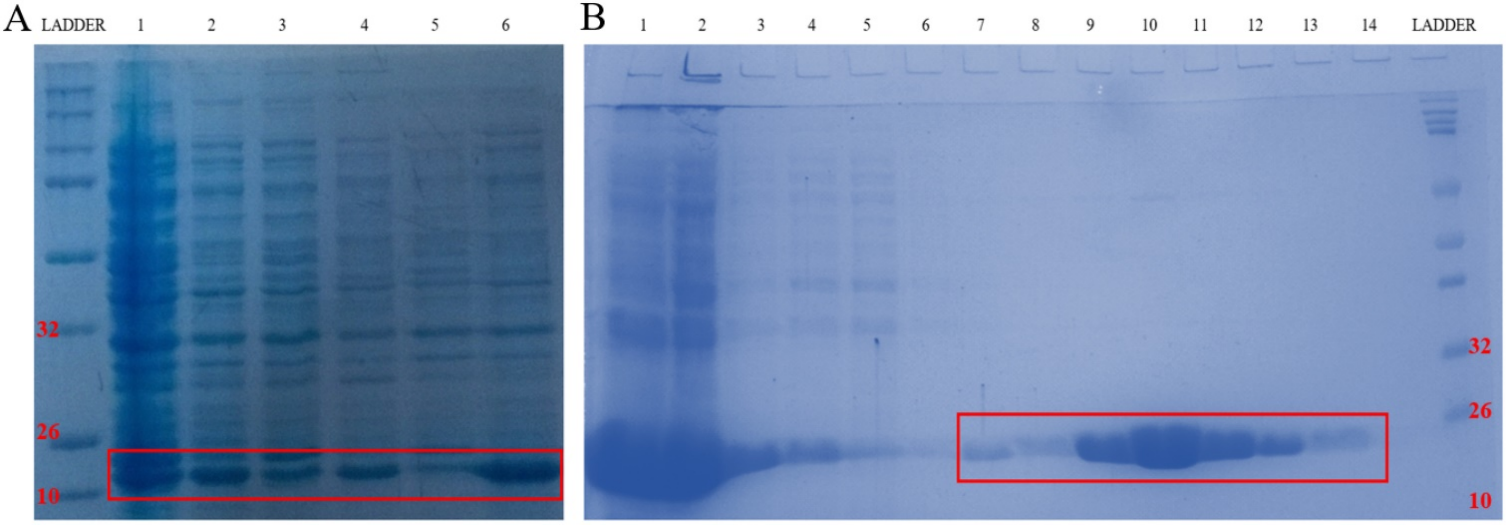
** A.** Codon Plus protein expression study on SDS page gel where Lane 1: un-induced @37°C for 1 h, Lane 2: IPTG induced @37°C for 1 h, Lane 3: un-induced @37°C for 3 h, Lane 4: IPTG induced @37°C for 3 h, Lane 5: un-induced @18°C overnight and Lane 6: IPTG induced @18°C overnight. **B.** Codon Plus protein purification study on SDS page gel where Lane 1: Cell Pellet, Lane 2: Before Sonication, Lane 3: Total Cell Lysate, Lane 4: Lysate supernatant, Lane 5: Lysate Flow Through, Lane 6: 10 mM Imidazole Wash, Lane 7: 25 mM Imidazole Wash, Lane 8: 150 mM Imidazole Elution 1, Lane 9: 150 mM Imidazole Elution 2, Lane 10: 150 mM Imidazole Elution 3, Lane 11: 250 mM Imidazole Elution 1, Lane 12: 250 mM Imidazole Elution 2, Lane 13: 250 mM Imidazole Elution 3, Lane 14: 250 mM column wash. The data shown is representative of three independent experiments carried out under the same conditions.

To further confirm that emodin and MTH1 binding occurs at the cellular level and is responsible for the augmentation of ROS, damaged dNTPs, and growing DNA damage in NSCLC cells, MTH1 protein was studied for its binding to emodin bio-physically. The plasmid containing the NUDT1 (N-terminal His-tagged) gene was revived from a DH5α bacterial stab and the NUDT1 plasmid was extracted with the plasmid purity determination by agarose gel (Supplementary Figure 5A). The plasmid was then transformed into *Escherichia coli* BL21-codon plus strain (Figure 15A).

After transformation and production, the protein content of the major purification steps at each level-the total cell lysate, the lysate supernatant, the flow-through, the wash, the matrix-bound, and the elution-were analyzed on a 12% sodium dodecyl sulfate polyacrylamide gel (SDS-PAGE) following Coomassie brilliant blue (CBB) staining. The purified protein concentration was determined using a NanoDrop spectrophotometer, measuring the absorbance A_280_. The final yield of purified MTH1 was approximately 0.65 g/l of culture (Figure 15B). To remove imidazole, the purified protein was dialyzed using 1X-PBS and concentrated in concentrator columns (5 kDa cutoff range).

### CD Spectroscopy Ascertaining the Folding Conformation of MTH1

**Figure 16:**
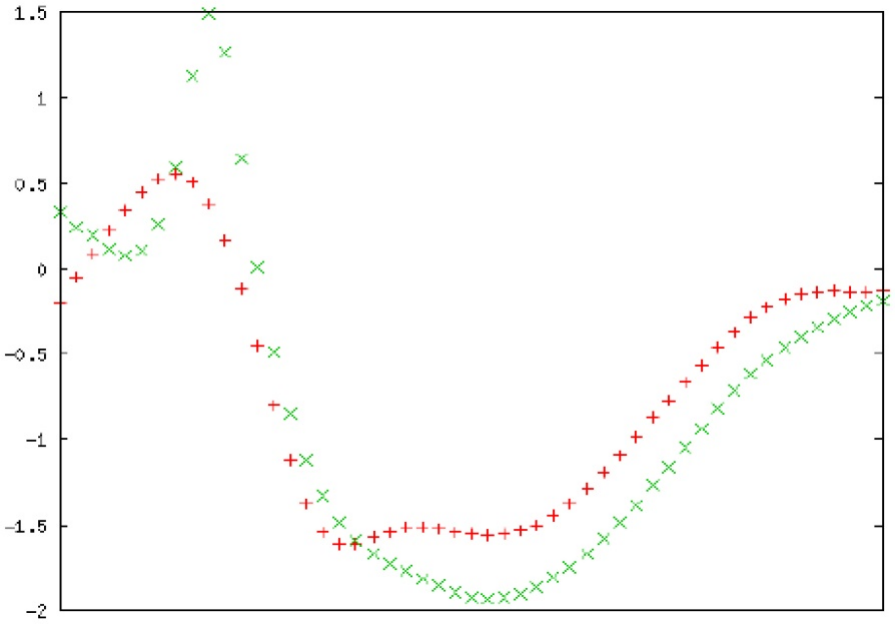
Secondary structure analysis by circular dichroism. Spectra of the MTH1 protein is presented in molar ellipticity (deg.cm^2^.dmol^-1^). The spectrum was recorded at 25 °C in a 0.01 cm path cell length quartz cell using MTH1 protein at a concentration of 5 µM in a 10 mM Tris-HCl buffer (pH 7.4). Red: input structure; Green: predicted structure. An average of three consecutive spectral scans was used and corresponding blank buffer was subtracted.

To confirm the folding of the purified MTH1 protein, circular dichroïsm (CD) spectrophotometric analysis was performed. The CD spectra of MTH1 were studied and the secondary structure was monitored. The signals recorded between 190 and 260 nm were superposed. Three consecutive spectral scans were averaged, the corresponding blank buffer was subtracted, and the data was expressed as the mean residue molar ellipticity, MRME (deg cm^2^/dmol). The secondary structure calculation was performed using K2D3 software (http://cbdm-01.zdv.uni-mainz.de/~andrade/k2d3/) for spectrum analysis to quantify the α helix, β sheets, and random coil content 47. The far-UV spectra of the proteins displayed an approximate 3.67% helical character and 35.98% beta sheet against an expected structure of 14% helical and 40% beta sheet (Figure 16).

### Bio-Physical Evaluation of Emodin Binding MTH-1 for Functional Inhibition

**Figure 17:**
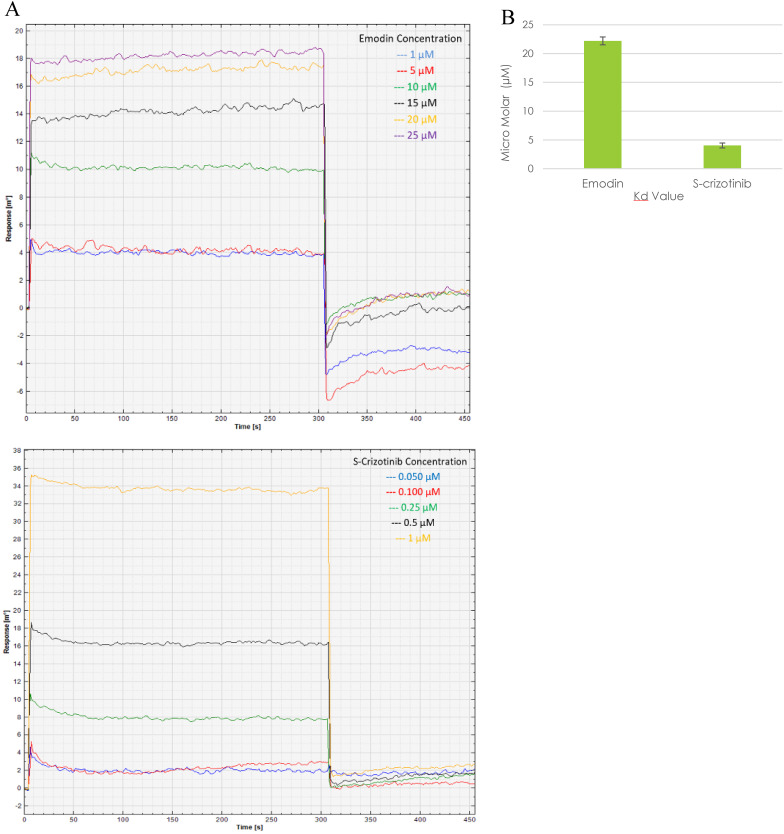
** Surface Plasmon Resonance sensorgram. A.** SPR sensorgram showing surface competition assay of MTH1 protein (ligand) with emodin and (S)-crizotinib (analytes). As the concentration of the analyte molecule increased, the SPR response increased. **B.** plot of the mean K_d_ values of MTH1 protein (ligand) with emodin and (S)-crizotinib (analytes). An average of three K_d_ values was taken for plotting the mean.

Presently, surface plasmon resonance (SPR) is one of the most widely used techniques for the biophysical assessment of small molecule interactions to develop target‐based drugs. The SPR assay was performed to assess the real time binding of MTH1 with emodin at physiologically relevant concentrations in real time, establishing their affinity. Being a surface technique, it allows for the performing of experiments at a much lower concentration range. Using MTH1 protein as a ligand (covalently coated to an activated monolayer of self-assembled 11-mercaptoundecanoic acid [MUA] on a gold surface, following the amine coupling method) and different concentrations of emodin and (S)-crizotinib as analytes, SPR was performed. The kinetic analysis sensorgrams (Figure 17A) showed a concentration dependent increase in the binding of emodin and (S)-crizotinib during the association phase. The binding affinity was found to be significant with a calculated KD value of 22.2 ± 0.68 µM for emodin and 4.04 ± 0.43 µM for (S)-crizotinib (positive control), ascertaining that emodin has a role in MTH1 inhibition as its highly probable interacting partner (Figure 17B).

### 8-OHdG quantification to assess DNA damage due to Emodin mediated MTH1 functional inhibition

**Figure 18:**
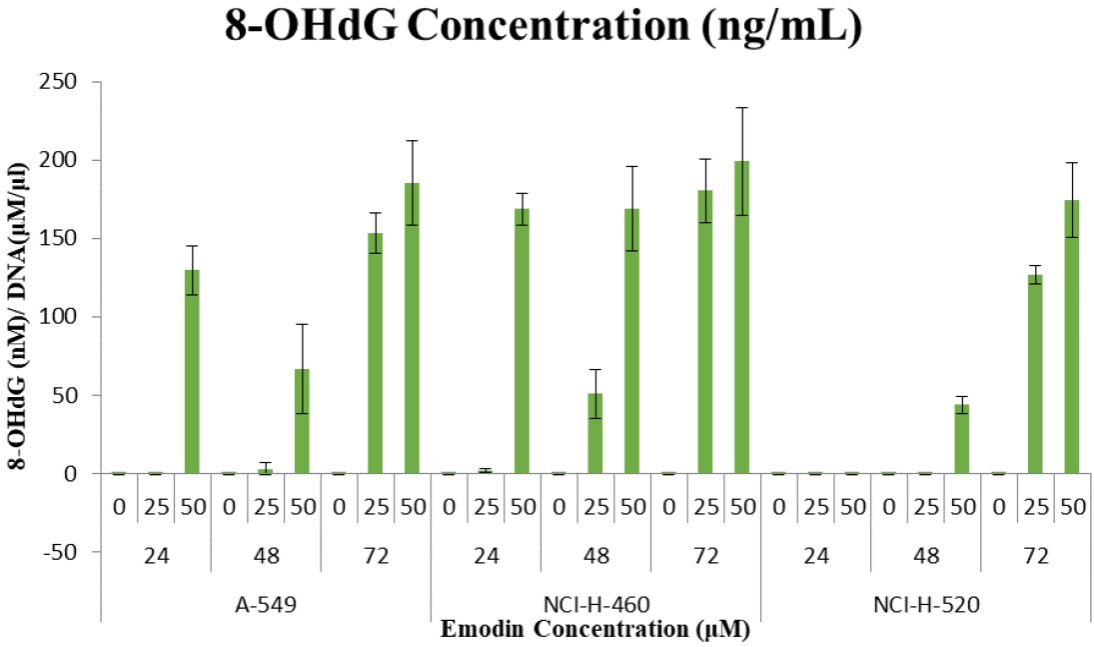
Quantification of 8-hydroxy-2'-deoxyguanosine (nM)/ DNA (µM/ml). A-549 (adenocarcinoma), NCI-H-460 (Large cell carcinoma) and NCI-H-520 (squamous cell carcinoma) cells under emodin treatment (25, 50 µM) at 24, 48 and 72 h along with vehicle controls respectively as quantified using 8-OH-dG ELISA protocol.

These observations that the DNA damage occurring due to emodin mediated MTH1 functional inhibition were substantiated by a considerable increase in the 8-OH deoxy-guanosine levels in the DNA from NSCLC cells, quantified by using ELISA. Emodin treatment resulted in a dose and time dependent increase in the concentration of 8-hydroxy-2'-deoxyguanosine (nM)/ DNA (μM/ml) in all the three NSCLC cell lines (Figure 18). In A-549 the concentration of 8-OH-dG showed an increase from approximately 130 nM to 185 nM from 24 to 72 h. Likewise in NCI-H-460, the concentration of 8-OH-dG showed an increase from approximately 160 nM to 200 nM from 24 to 72 h. In NCI-H-520 the concentration of 8-OH-dG showed an increase from approximately 140 nM to 175 nM from 24 to 72 h. Taken together, emodin treatment thus induces MTH1 functional inhibition and augments the concentration of oxidized dNTPs, enhancing DNA oxidative damage and subsequent cancer cell death.

## Discussion

Interestingly, the MTH1 protein is essential to efficient survival in cancer cells but its function is not crucial for normal cells, creating research interest in developing anti-cancer strategies based on MTH1 inhibition. Cancer cells are marked by dysfunctional redox regulation. This accumulation of ROS creates a cellular environment of oxidized nucleotides that make it essential for the MTH1 protein function to prevent their mis-incorporation and subsequent DNA damage during augmented replication and rapid division. MTH1 is overexpressed in several cancers and its catalytic function is noticeably augmented in lung cancers as a non-oncogene addiction function and a response to survive by circumventing the nuclear incorporation of damaged dNTPs 22. Since current anti-cancer approaches are dominated by target proteins essential to normal cells, causing severe side effects when treated with chemotherapies, MTH1 inhibition is a promising anti-cancer approach. The current work aims to identify novel natural small molecular inhibitors to the Nudix hydrolase protein (MTH1) to be used as an anti-cancer therapeutic.

Natural compound libraries were screened to obtain MTH1 active site binding moieties and validated the most promising, emodin, for its effect in NSCLC cell line models upon MTH1 inhibition. It was validated that emodin treatment on NSCLC caused a surge in ROS levels, loss of mitochondrial potential, genome-wide double-stranded DNA damage, and apoptosis. Emodin caused a dose and duration dependent decline in the proliferation of NSCLC cells, a G2/M phase cell cycle arrest, decreased in the cyclin-D1, CDK-2, and CDK-4 and an increase in cyclin-B1 protein levels. There was also a rise in the number of apoptotic cells, protein level expression profiles of Bax (pro-apoptotic), survivin and decrease in Bcl-2 (anti-apoptotic) in a duration dependent manner. The cleavage of caspase-3 and PARP increased, as did the p-21 levels. Emodin caused an increase in mitochondrial and cytoplasmic ROS levels and significantly reduced the mitochondrial membrane potential. Emodin caused extensive DNA damage and fragmentation, with an increase in the repair protein levels ATM-p, DNA-PKCs, and 53 BP-1. We observed the mechanism of emodin action: augmented ROS levels causing extensive dNTP oxidation, subsequent DNA damage, and apoptosis via MTH1 inhibition. Since the DNA damaging effects of emodin treatment were reported earlier 29, 37 but its mechanism of action had not been elucidated clearly, this paper highlights for the first time that the effect of emodin is via the mechanism of MTH1 inhibition. We expressed the human MTH1 protein in an *E. coli* bacterial system to perform binding analysis of emodin with MTH1 protein using SPR studies. Emodin binds MTH1 in a concentration dependent manner, proving the genotoxic and cytotoxic effects of emodin are due to its capability to inhibit MTH1 function in cancer cells.

The effect of emodin highlights a novel concept to exploit the unique oxidative status of tumors, increase ROS, and concurrently target the redox-protective MTH1 function for cancer therapy. Thus, the mode of emodin targeting the nucleotide pool homeostasis is a promising cancer cell specific natural small molecule therapeutics.

## Conclusion

The tumor specific unique redox status makes the rapidly proliferating cells more dependent on redox protective mechanisms such as MTH1 upregulation, opening a novel therapeutic window for the selective induction of cancer cell apoptosis via oxidative stress overload, sparing the normal cells. The dual dNTP pool targeting of emodin (increasing ROS on one hand and inhibiting oxidized dNTP pool sanitation by MTH1 on other) shows an additive effect on apoptotic cancer cell death, highlighted by augmented DNA damage and corresponding ATM repair pathway upregulation. Conclusively, we demonstrate that MTH1 protein targeting using a novel natural small molecular inhibitor, emodin, constitutes a simple and safe anti-cancer therapeutic approach that aims to enhance the deregulated redox metabolism and increase ROS levels in cancer to cause extensive DNA damage specific to cancer cells and therefore leading to senescence.

## Supplementary Material

Supplementary materials and figures.

## Figures and Tables

**Table 1:** Measurement of the Binding affinity scores, energies, molecular interactions as well as H-bond distances of the highest scoring docked compounds with MTH1 using XP docking protocol

Compound	Glide score (XP) (kcal/mol)	Interacting residues	Distance (H-bond, Å)	Glide energy (kcal/mol)
Ligand_71308639 (Taxifolin hydrate) (Sigma Phytochemical compounds library)	-8.76	Gly28, Asn33, Asp119, Thr8, Lys23, Phe27, Arg31, Asn33, Gly 34, Phe72, Met81, Trp117.	2.95 3.28 3.30	-43.26
Ligand_148 (Quercetin dihydrate) (Natural remedies compound Library)	-8.74	Asn33, Gly36, Leu9, lys23, Phe27, Gly 34, Phe35, Glu56, Phe72, Met81, Trp117, Asp119, Asp120, Phe139.	3.21 3.01	-45.37
Ligand_3220 (emodin) (Sigma Phytochemical compounds library)	-7.63	Gly36, The8, Leu9, lys23, Phe 27, Gly 28, Asn33, Gly37, Ile70, Phe72, Val83, Trp117, Asp119, Try123.	3.22	-29.43

**Table 2:** Measurement of the Docking scores of lead compounds with MTH1 using AutoDock 4.0.1 software protocol

Compound	Emodin	Taxifoline hydrate	Quercetine Dihydrate
Docking Score	-6.83	-5.77	-5.81

**Table 3:** Measurement of the Molecular mechanics energy in combination with the generalized Born or Poisson-Boltzmann and surface area solvation (MMPBSA) parameters

MMPBSA (Kcal)	MM (Kcal)	PB (Kcal)	SA (Kcal)	Vdw (Kcal)	Elec (Kcal)
-167.8995024	-425.5168452	271.5267	-13.9094	-168.68	-256.837
