# Supplementary material for: A Double-Edged Sword: The Anti-Cancer Effects of Emodin by Inhibiting the Redox-Protective Protein MTH1 and Augmenting ROS in NSCLC
Source: J Cancer. 2021 Jan 1;12(3):652–81. doi: 10.7150/jca.41160 (PMC7778552; doi:10.7150/jca.41160)
Supplement: Supplementary file 1 — Supplementary materials and figures. [file jcav12p0652s1.pdf]

Supplementary Materials and Methods

Screening of MTH-1 inhibitors

Protein preparation

The native human MutT Homolog 1 (MTH1) protein X-ray crystallography resolved structure, having 8-oxo-dGTPase function (Nudix hydrolase family) (PDB ID: 3Q93), with a resolution of 1.8 Å was retrieved for the study from the RCSB protein repository known as the Protein Data Bank (PDB) [1]. The PDB structure contained 154 amino acids from 3-156 residues along with water. For all subsequent analysis, the water molecules contained in the structure, were removed using the Accelrys Viewerlite 5.0 software [2]. The retrieved protein structure was next pre-processed, adjusted and optimized for further analysis with the aid of the inbuilt Protein Preparation Wizard embedded in software Schrodinger Suite, Maestro interface for docking studies [3]. The 3D structure processing involved the addition and optimization of hydrogen bonds by fixing bond orders, bond lengths, removal of bad contacts and steric clashes, creation of disulfide bonds and stabilizing ionization states, protein terminal capping, conversion of the seleno-methionine residues to methionine and fixing of the missing amino acids and side-chain orientations. The prepared MTH1 structure was further optimized to acquire an energetically minimized conformation using the OPLS (Optimized Potentials for Liquid Simulations) force field [4]. Further flexible filtering was applied for discarding the ligands with poor pharmacological characteristics by applying the Lipinski's filter [5].

Ligand preparation

For the screening purposes, we created a virtually available library of ligands by extracting the natural compounds from the freely available special subsets of two libraries, first, the phytochemical compound library from compounds available at Sigma and Natural Remedies. For the docking approach, the libraries were prepared and processed using the Schrodinger Suite, LigPrep Wizard [6]. For inclusion of all ligand conformations, all the possible stereochemical, tautomeric or ionization variants of the ligand molecules were created for screening and subsequent energy minimization.

Grid generation

With the aid of the Receptor Grid generation panel available in the Glide docking module, Schrodinger suite, a grid using the centroid of the following residues: Leu9, Phe27, Asn33, Phe72, Met81, Val83, Trp117, Asp119, Asp120, Trp123 and Phe139, was generated with a radius of 20Å. The parameters such as the scaling factor was kept at 1.0, the partial charge cut-off was set at 0.25 (pre-set values) and no additional constraint values were used [7-10].

Virtual screening: The high throughput virtual screening (HTVS) and extra precision (XP) docking

The growing need of efficient and rapid search for novel binding partners to proteins, has paved the way for the emergence of Virtual screening as an extremely effective filtering tool for the process of computer aided drug-designing. It has been extensively applied for computing of the binding affinities and elucidation of the binding modes for numerous ligand receptor complexes.

For lead ligand identification, the prepared ligand library was docked into the active site of the MTH1 protein employing the Schrodinger's Glide module also known as the Grid-based Ligand Docking along-with its Energetics [7, 9]. The Glide algorithm is derived from a systematic virtual screening protocol using the searching algorithm based on incremental construction for creating an output called as the G-Score scoring function, combined with a series of parameters [11]. Computationally, Glide performs a ligand specific extensive search for the conformation, location and orientation binding parameters over the protein active site to generate an output in terms of the Glide Score (GScore) and Glide energy.

A two-step docking strategy for performing the virtual screening was employed: The high-throughput virtual screening (HTVS) and the extra precision (XP) docking employing the Glide virtual screening workflow (GVSW) [7]. The essential grid coordinates were first employed to perform the MTH1 inhibitor screening using the HTVS docking algorithm of Glide [9]. A rational virtual screening workflow from HTVS to XP is performed using Glide, obtaining enriched data at each level to shortlist fewer compounds for studying higher precision levels. A docking Score of -7kcal/mol threshold was set for compound screening and for obtaining higher precision docking, the compounds above the threshold values were subsequently subjected to the extra precision Glide (XP), that is a higher accuracy docking algorithm that ensues more advanced screening [11]. The three natural compounds with top docking scores with MTH1were selected and their results are tabulated in Table 1.

Validation of docking by AutoDock

The docking results from the Glide module were subjected to further confirmation using the AutoDock software Molecular Docking Suite version 4.0, using the same crystal structure as above [12]. The missing atoms were repaired, heteroatoms were removed, polar hydrogens were added for ionization correction and amino acid residue tautomeric states and the non-polar hydrogens were merged from the protein for its preparation for Autodock. For the generation of energy based AutoDock scoring function, hydrogen bonding, entropy losses, electrostatic and van der Waals forces were studied [12, 13]. The parameters of Gasteiger charges and rigid roots were allocated to the lead ligands and scoring grid of the energy function of 60 Å × 60 Å × 60 Å (x, y, z) dimensions was generated for all the key residue incorporation. The adaptive local searching protocol of the default parameters of the Lamarckian genetic algorithm were used to identify a maximum fitting conformation.

Molecular dynamics

Molecular dynamics simulations of docked complex

The structure of MTH1 protein bound to the lead compounds was analyzed using the Ligplot program for hydrogen bonding and other non-bonded interaction calculation between the atoms of ligand and protein [14]. Also, the three-dimensional structural models of the complex of MTH1 protein bound to the lead compounds were viewed in the PyMOL program to study how well the compounds were fitting inside the protein active site.

We further employed Molecular dynamics (MD) simulation as a protocol to perform further stability study of the lead compound-MTH1 binding. The native MTH1 protein was used for docking with the lead compound, emodin and the energy minimized stable structure was used for performing MD simulation studies for 50 ns time. The simulation was run using the GROMACS software version 5.0, employing the GROMOS96 43a1 force field in a cubic box

with water model spc [15, 16]. For the Gromos force field, iGROMACS compatible ligand topologies were created with the help of the PRODRG server [17]. The system underwent energy minimization up till a maxima of <10.0 kJ/mol/nm was achieved at 50,000 ns steps, using the steepest descent algorithm. The LINCS algorithm was used for bond lengths constraining [18]. The unfavorable contacts between molecules were relaxed using the Verlet cut-off scheme. Molecular dynamics was run on the equilibrated system for 50 ns along with time-step integration of 2 fs employing the leap-frog integrator. The MD trajectory frames were saved for every 10 ps. The cluster analysis approach representative protein frame achieved from the 50 ns trajectory was quality assessed for geometrical and energy parameters. The trajectory files were analyzed using numerous statistical parameters of the various inbuilt scripts of GROMACS.

RMSD and RMSF Analysis

The RMSD (root mean square deviation) analysis is used to understand the ligand-protein complex stability and simulation convergence time. The RMSF (root mean square fluctuation) value noticeably illustrates the protein segment residue flexibility differences that correlate to the numerous intermolecular hydrogen bonding interactions and contacts of hydrophobic nature identified along the analysis. The RMSD and RMSF values of the MTH1-emodin complex were calculated taking reference of the first frame along the whole simulation trajectory.

Radius of gyration

The atomic distribution from the center of mass that is common to all molecules, calculated as the mass-weighted root mean square distance ($RMSD_{mw}$) in the system is called as the radius of gyration. Any protein mutation can cause an increase or decrease in protein Rg as each protein comprises of its own characteristic radius of gyration. The Radius of gyration is also affected after favorable ligand binding as it determines the protein compactness and overall protein complex dimensions, responsible for their respective interactions. The radius of gyration for emodin-MTH1 complex was computed throughout the 50 ns trajectory.

H-Bond Length analysis

Hydrogen bonds also play a crucial role in the protein structure stability. The protein-drug complex intermolecular H-bonding pattern along with other interactions was studied for 50 ns simulation and analyzed describing the complex stability. The MD simulations of 50 ns reveal significant outcomes reflecting the structural stability, protein dynamics and interactions for the binding mechanism elucidation.

MMPBSA binding free energy calculation

MMGBSA and MMPBSA (Molecular mechanics energy with generalized Born or Poisson–Boltzmann and surface area solvation) methodology is regularly being implied estimation of the biological macromolecule and small ligand free interaction energies [19]. Considering the free and interacting state free energy differences of the two moieties to provide comparative free energies for them in distinct solvated conformations, comprises the main objectives of these approaches. The binding free energies for the Protein drug interaction were computed using the MMPBSA methodology, to create accurate calculations of the drug binding affinity relative to its target proteins.

The g_mmpbsa package was used to calculate the binding free energy of emodin, employing the MMPPSA method. Here, the $\Delta G_{binding}$ is computed from free energies of the receptor ligand complex with the equation:

$\Delta G_{binding} = G_{complex} - G_{ligand} - G_{receptor}$

For the emodin- MTH1 complex, a 50 snapshot trajectory file was used in a 200 frame time-step for estimation of the free binding energy, electrostatic energy, Van der Waals (VDW), polar solvation and the solvent-accessible surface area (SASA) energy.

Assessment of cell morphology

NSCLC cells were cultured until 80% confluence, then seeded in 24 well plates in 500 μL of RPMI medium at a cell density of approximately 10^5 cells/well. The cells were treated with emodin at different dilutions (0, 25, 50 and 75 μM) in dimethyl sulfoxide (DMSO), DMSO vehicle < 0.5% volume) for 24, 48 and 72 h. Then, post treatment the cells were examined under a phase-contrast microscope (Zeiss Primovert) and photographed.

Assay of cell growth and Viability: IC_{50} determination

Trypan Blue dye exclusion Assay:

NSCLC cells were cultured until 80% confluence, then seeded in 24 well plates in 500 μL of RPMI medium at a cell density of approximately 10^5 cells/well. The cells were treated with emodin at different dilutions (0, 1, 10, 25, 40, 50 and 75 μM) in DMSO (vehicle < 0.5% volume) for 24, 48 and 72 h. Then, post treatment the cells were centrifuged, washed with PBS and then suspended in PBS (pH 7.4) containing 0.4% trypan blue solution.

The percent live and dead cells was determined using a haemocytometer for counting the cells stained with trypan blue. The cells with a properly defined cellular outline, excluded the dye and were scored as alive. Whereas the cells not excluding it were scored as dead. The total cell number, live cell number and percentage of dead cells was calculated from three independent experiments.

$$\text{Total cell count} = \text{Total Live cell count} + \text{Total Dead cell count}$$

$$\%\ \text{Dead cells} = (\text{Total Dead cell count} / \text{Total cell count}) * 100$$

MTT Assay:

NSCLC cells were cultured until 80% confluence, then seeded in 96-well plates in 100 μL of RPMI medium at a cell density 10^4 cells/well. The cells were then treated with emodin at different dilutions (0, 10, 25, 50, 75, 100, 150 and 200 μM) in DMSO (vehicle < 0.5% volume) for 24, 48 and 72 h.

After incubation, the treatment containing media was removed and 100 μL of working 3-(4, 5-dimethylthiazol-2-yl)-2, 5-diphenyltetrazolium bromide (MTT) stock solution (0.5 mg/mL prepared culture media) was added to each well and the cells were incubated for 3 h in a humidified atmosphere containing 5% CO_2. The MTT containing media was carefully aspirated, without disturbing the adherent cells and the formazan that formed was solubilized from the cells by adding 100 μL of DMSO to each well. The colour intensity of formazan was measured by the absorbance at 595 nm with a 96-well ELISA plate spectrophotometer

(2300 EnSpire Multimode Plate Reader, PerkinElmer). The rate of tetrazolium reduction to formazan is directly proportional to the rate of cell proliferation and subsequent cell viability. MTT assays were performed in triplicate and each measurement contained three parallels (n=9).

$$\% \text{ inhibition} = 100 - 100 \times (A_{595nm}) \text{ treated} / (A_{595nm}) \text{ control}$$

$$\% \text{ cell viability} = 100 - \% \text{ inhibition}$$

Wound- healing Assay

NSCLC cells were cultured until 80% confluence, then seeded in 6 well plates in 3ml of RPMI medium at a cell density of approximately 10^5 cells/ml and grown till the formation of a confluent monolayer. The confluent cell monolayer was scraped using a p200 pipet tip in a straight line to create a “scratch/wound”. The cells were washed to remove debris with 1 ml RPMI and fresh 3ml RPMI media was added for the *in vitro* scratch/wound assay. The cells were subsequently treated with emodin at different dilutions (0, 25, 50 and 75 μM) in DMSO (vehicle < 0.5% volume) for 24, 48 and 72 h. Then, post treatment the cells were washed (2 times) with PBS and observed under phase-contrast microscope (Zeiss Primovert) and photographed. The images were analysed using ImageJ software (Wound Healing Tool).

Mitochondrial membrane potential (ΔΨM) assay

JC-1 and $DIOC_6$ [3] iodide dyes: Microscopic Assessment of Mitochondrial membrane potential

NSCLC cells were cultured until 80% confluence, then seeded on coverslips in 24 well plates in 500 μL of RPMI medium at a cell density of approximately 10^5 cells/well. Then they were treated with emodin at different dilutions (0, 1, 10, 25, 40, 50 and 75 μM) in DMSO (vehicle < 0.5% volume) for 24, 48 and 72 h. Carbonyl cyanide m-chlorophenyl hydrazone (CCCP), that functions as a mitochondrial uncoupler and a chemical inhibitor of oxidative phosphorylation causing mitochondrial depolarization, was used as a positive control at a concentration of 50 µM, for 10 min. Post- treatment, the media above the coverslips was aspirated and 300 µl of fresh media containing - 1µg/ml JC-1 (working concentration from 1mg/ml JC-1 in DMSO stock solution) or 1µM $DIOC_6$ [3] iodide (working concentration from 1mM $DIOC_6$ [3] iodide in DMSO stock solution) added to each well. Then cells on coverslips were incubated at 37°C in humidified 5% CO_2 chamber for 20 min. Post incubation they were washed (2 times) with PBS (pH 7.4) and stained with a 2µg/ml working Hoechst staining solution from a Hoechst 10 mg/mL stock in PBS for 10 min at room temperature, in dark. The cells were washed (2 times) with PBS and the coverslips were mounted in 1,4-diazabicyclo [2.2.2] octane (DABCO) (Sigma Aldrich) Mounting Medium (containing 1% DABCO (w/v) in 80% glycerol and 20% PBS). The coverslips were acquired immediately and analyzed with a Nikon Real Time Laser Scanning Confocal Microscope at 60X magnification with supporting software.

Nuclear morphological Assessment (Hoechst 33342 staining)

NSCLC cells were cultured until 80% confluence, then seeded on coverslips in 24 well plates in 500 μL of RPMI medium at a cell density of approximately 10^5 cells/well. The cells were treated with emodin at different dilutions (0, 25, 50 and 75 μM) in DMSO (vehicle < 0.5% volume) for 24, 48 and 72 h. Post treatment the cells were washed (2 times) with PBS and

stained with a 2µg/ml working Hoechst staining solution from a Hoechst 10 mg/mL stock in PBS for 10 min at room temperature, in dark. The cells were washed (2 times) with PBS and fixed with 4% paraformaldehyde solution for 15 min at room temperature. Post fixing, the cells were washed (2 times) with PBS and the coverslips were mounted on DABCO (Sigma Aldrich) Mounting Medium (containing 1% DABCO (w/v) in 80% glycerol and 20% PBS). The Images were acquired and analysed with a Nikon Real Time Laser Scanning Confocal Microscope at 60X magnification with supporting software.

Anenexin V/PI Apoptosis Analysis

Microscopic Assessment of Apoptosis

For confocal microscopy, NSCLC cells were cultured until 80% confluence and seeded on coverslips, placed in 24-well plates at a cell density of approximately 10^4 cells/well and the cells were treated with emodin at different dilutions (0, 25, 50 and 75 µM) in DMSO (vehicle < 0.5% volume) for 24, 48, 72 h; following which the cells were washed (2 times) with PBS. The cells on coverslips were then stained with 5 µL Alexa Fluor® 488 annexin V and 1 µL 100 µg/mL PI working solution (PI) per 100 µL in each well, in dark at room temperature for 15 min, according to the manufacturer's instruction (Dead Cell Apoptosis Kit with Annexin V Alexa Fluor™ 488 & Propidium Iodide (PI), Thermo Fisher Scientific). After a PBS wash, the DNA was counterstained with a 2µg/ml working Hoechst staining solution from a Hoechst 10 mg/mL stock in PBS for 10 min at room temperature and the coverslips were mounted in DABCO (Sigma Aldrich) Mounting Medium (containing 1% DABCO (w/v) in 80% glycerol and 20% PBS). Immediately, images were acquired using fluorescence emission at 530 nm and >575 nm and analysed with a Nikon Real Time Laser Scanning Confocal Microscope at 60X magnification with supporting software.

Cell lysate Preparation

NSCLC cells were grown in T_{25} flasks to 60% confluence. Then after 18 h, the cells were treated with emodin at 50 µM dilution in DMSO (vehicle < 0.5% volume) or vehicle alone for 24, 48, 72 h. After treatment both the floating and adherent cells were collected and washed twice with PBS. The cells were then pelleted down at 1000 rpm for 3 min. 100µl of lysis buffer (25mM Tris, pH 7.4, 150 mM NaCl, 0.1% SDS, 1% Nonidet P-40, Protease inhibitor cocktail (Sigma Aldrich)) was added to each sample and the pellet was resuspended. The samples mixed with lysis buffer were kept on ice for 30 minutes with gentle intermittent tapping. The after which the samples were pelleted at 15000 rpm for 45 min at 4˚C. The supernatant was collected and used as the total cell lysate.

Measurement of intracellular ROS

Microscopic Assessment of ROS

For ROS level measurement or oxidative stress detection NSCLC cells were cultured until 80% confluence and seeded on coverslips in 24 well plates in 500 µL of RPMI medium at a cell density of approximately 10^5 cells/well. The cells were treated with emodin at different dilutions (0, 1, 10, 25, 40, 50 and 75 µM) in DMSO (vehicle < 0.5% volume) for 24, 48 and 72 h. CCCP was used as a positive control at a concentration of 50 µM, for 10 min. Post-treatment, the media above the coverslips was aspirated and 300 µl of fresh media containing 5 µM CellROX™ Deep Red Reagent, Sub-Cellular Localization: Cytoplasm (Thermo Fisher

Scientific), (working concentration from 2.5 mM CellROX™ Deep Red Reagent stock solution) was added to each well. The 24 well plate containing coverslips was incubated at 37°C in humidified 5% CO_2 chamber for 30 min. Post incubation, the cells were washed (2 times) with PBS (pH 7.4) and fixed with 4% paraformaldehyde in PBS for 20 min. After fixing, the cells were rinsed with PBS and stained with a 2µg/ml working Hoechst staining solution from a Hoechst 10 mg/mL stock in PBS for 10 min at room temperature, in dark. The cells were washed (2 times) with PBS and the coverslips were mounted on DABCO (Sigma Aldrich) Mounting Medium (containing 1% DABCO (w/v) in 80% glycerol and 20% PBS). The coverslips were acquired at absorption/emission maxima of 644/665 nm and analyzed with a Nikon Real Time Laser Scanning Confocal Microscope at 60X magnification with supporting software.

Similarly, staining was done with CellROX™ Green Reagent (Thermo Fisher Scientific), for oxidative stress detection having a Sub-Cellular Localization: at the level of Mitochondria and Nucleus.

Fluorometric Assessment of ROS

For ROS level measurement, NSCLC cells were cultured until 80% confluence, then seeded in 96 well plates in 100 µL of RPMI medium at a cell density of approximately 10^3 cells/well. The cells were treated with emodin at different dilutions (0, 1, 10, 25, 50, 75, 100 and 200 µM) in dimethyl sulfoxide (DMSO), DMSO vehicle < 0.5% volume) for 24, 48 and 72 h. CCCP was used as a positive control at a concentration of 50 µM, for 10 min. Post-treatment, the media was aspirated and 100 µl of fresh serum free media containing 5 µM CellROX™ Deep Red Reagent, Sub-Cellular Localization: Cytoplasm (Thermo Fisher Scientific), (working concentration from 2.5 mM CellROX™ Deep Red Reagent stock solution) was added to each well. The 96 well plate was incubated at 37°C in humidified 5% CO_2 chamber for 30 min. After incubation the CellROX™ Deep Red Reagent dye fluorescence was acquired with a 96-well ELISA plate-reader (2300 EnSpire Multimode Plate Reader, PerkinElmer) using wavelengths of absorption/emission maxima of 644/665 nm. The staining was performed in triplicate and each measurement contained three parallels (n=9).

Similarly, staining was done with CellROX™ Green Reagent (Thermo Fisher Scientific), for oxidative stress detection having a Sub-Cellular Localization: at the level of Mitochondria and Nucleus, at absorption/emission maxima of 485/520 nm.

DNA Fragmentation Analysis

NSCLC cells were seeded in 6 well plates at a cell density of approximately 10^5 cells/well and treated with emodin at different dilutions (0, 25, 50 and 75 µM) in DMSO (vehicle < 0.5% volume) for 24, 48 and 72 h. Post treatment the adherent and floating cells were collected and washed with PBS. The cell pellet was used to extract the genomic DNA using Genomic DNA Miniprep Kit (MDI membrane technologies) as per manufacturer’s protocol. DNA was then resuspended in Tris EDTA buffer (pH 8.0). The concentration of the genomic DNA was determined by measuring the absorbance A_{260} in NanoDrop Spectrophotometer and equal concentration of the obtained genomic DNA from each sample was loaded onto an 1.2 % agarose gel, containing ethidium bromide (0.1 mg/ml), electrophoresed at 80 V. DNA fragmentation pattern was observed using the Syngene G:Box Gel Doc.

Indirect Immunofluorescence

For confocal microscopy, NSCLC cells were seeded on coverslips, placed in 24-well plates at a cell density of approximately 10^4 cells/well and the cells were treated with emodin at 50 μM dilution in DMSO (vehicle < 0.5% volume) or vehicle alone for 24, 48, 72 h; following which the cells were washed (2 times) with PBS (PBS) and fixed with 4% paraformaldehyde in PBS for 20 min. These fixed cells were then washed with PBS and permeabilization was done with 0.5% Triton-X-100 for 5 min. The PBS washed slides were then incubated for 1 h with 5% BSA (bovine serum albumin) in PBS following which cells were stained with various primary antibodies for 3 h. After rinsing with PBS, the coverslips were incubated with Secondary Antibody for 1h (diluted 1:400) in 10% BSA in PBS. After PBS wash, the DNA was counterstained with a working Hoechst staining solution at a dilution of 1:2000 (Hoechst 10 mg/mL stock in PBS) at room temperature for 10 min and the coverslips were mounted on 1,4-diazabicyclo [2.2.2] octane (DABCO) (Sigma Aldrich) Mounting Medium (containing 1% DABCO (w/v) in 80% glycerol and 20% PBS). The Images were acquired and analysed with a Nikon Real Time Laser Scanning Confocal Microscope at 60X magnification with supporting software.

Bacterial Expression and purification of MTH-1

NUDT1 was a gift from Nicola Burgess-Brown (Addgene plasmid # 74660). The plasmid 74660 for NUDT1 gene (containing N-terminal His-tagged MTH1) was obtained as a bacterial stab of DH5α cells. The stab was revived, cells were used to prepare glycerol stocks and the NUDT1 plasmid was extracted using pDNA Extraction kit (MDI membrane technologies) and plasmid purity was determined by running on an agarose gel. The plasmid was then transformed into *Escherichia coli* BL21-CodonPlus competent cells and plated on LB Kanamycin agar plates to obtain single colonies. The transformed cells were grown in 100 ml cultures and Plasmid purification was done to give for sequencing and confirmation of the NUDT1 gene. Sequence homology with NUDT1 gene was obtained. The BL21-CodonPlus clone was grown in culture volumes of 100 ml in baffled flasks at 37 °C at 200 rpm. Once A_{600} nm reached 0.8, isopropyl-β-D-thiogalacto pyranoside (IPTG) (1mM) was added and protein expression profile was studied for 1h and 3h at 37˚C and overnight at 18˚C. Since maximum protein was obtained in the overnight cultures at 18˚C, large-scale expression and purification of MTH1 was done using the same protocol. The His-tagged MTH1 protein was purified from the bacterial lysate using Nickel- nitrilotriacetic acid (Ni-NTA Agarose) affinity chromatography Resin (30210, Qiagen, Germany). Ni-NTA column was washed with buffer A (10mM Tris-HCl pH 7.4, 1mM DTT, 500mM NaCl) and protein was eluted by buffer A fortified by 150mM imidazole. Samples from each purification step was analysed using SDS-PAGE followed by Coomassie staining. The concentration of the purified protein was determined with a NanoDrop Spectrophotometer by absorbance A_{280} measurement. Pure protein was stored at −80 °C for further experiments.

Circular Dichroism Spectroscopy

The CD spectra of MTH1 was studied on a JASCO J-815 spectro-polarimeter (JASCO Corporation, Tokyo, Japan) using a 1 mm path length cylindrical quartz cell. The MTH1 secondary structure was monitored in the region between 190 and 260 nm (far UV). An average of three consecutive spectral scans was averaged and corresponding blank buffer was subtracted. Data was expressed as the mean residue molar ellipticity (MRME) (deg

$cm^2/dmol$), which is defined as the CD corrected for concentration and expressed by the equation:

$$MRME = \frac{M \times \theta_\lambda}{10 \times d \times C \times r}$$

Where M is called as the molecular weight of the protein, Θ_λ is the CD ellipticity (mdeg), d is the path length (cm), C is the protein concentration (mg/mL) and r is defined as the number of amino acid residues in the protein. The secondary structure was calculated using the K2D2 software (http://cbdm-01.zdv.uni-mainz.de/~andrade/k2d3/) for the analysis of the spectrum obtained to quantify the content of α helix, β sheets and random coils [20].

SUPPLEMENTARY INFORMATION

Antibodies used: mouse anti - p53BP1 (pS25) (sc-135748, Santa Cruz Biotech), mouse anti - pATM (pS1981) (sc47739, Santa Cruz Biotech), mouse anti - DNA-PKcs (sc-390698, Santa Cruz Biotech), mouse anti - α-tubulin (sc-8035, Santa Cruz Biotech), mouse anti - β-Actin (sc-47778, Santa Cruz Biotech), mouse anti - survivin (sc-17779, Santa Cruz Biotech), rabbit anti - Bax (sc-493, Santa Cruz Biotech), mouse anti - Bcl-2 (sc-7382, Santa Cruz Biotech), mouse anti-Cdk2 (sc-6248, Santa Cruz Biotech), rabbit anti - Cdk4 (sc-601, Santa Cruz Biotech), rabbit anti - cyclin B1 (sc-752, Santa Cruz Biotech), rabbit anti - cyclin D1 (sc-753, Santa Cruz Biotech), mouse anti p - 21 (sc-6246, Santa Cruz Biotech), mouse anti - Integrin β1 (sc-8978, Santa Cruz Biotech), mouse anti - vimentin (sc-6260, Santa Cruz Biotech), goat anti-mouse IgG-HRP (sc-2005, Santa Cruz Biotech), goat anti-rabbit IgG-HRP(sc-2004, Santa Cruz Biotech), rabbit anti-p53 pS15 (9284, Cell Signaling Technologies), rabbit anti-Caspase-3 (full length and cleaved) (9662, Cell Signaling Technologies), rabbit anti-PARP (full length and cleaved) (9542, Cell Signaling Technologies), Goat anti-Mouse IgG Alexa Fluor-488 Secondary Antibody (A11001, Thermo Fisher Scientific), Goat anti-Rabbit IgG Alexa Fluor-488 Secondary Antibody (A11008, Thermo Fisher Scientific).

SUPPLEMENTARY FIGURES

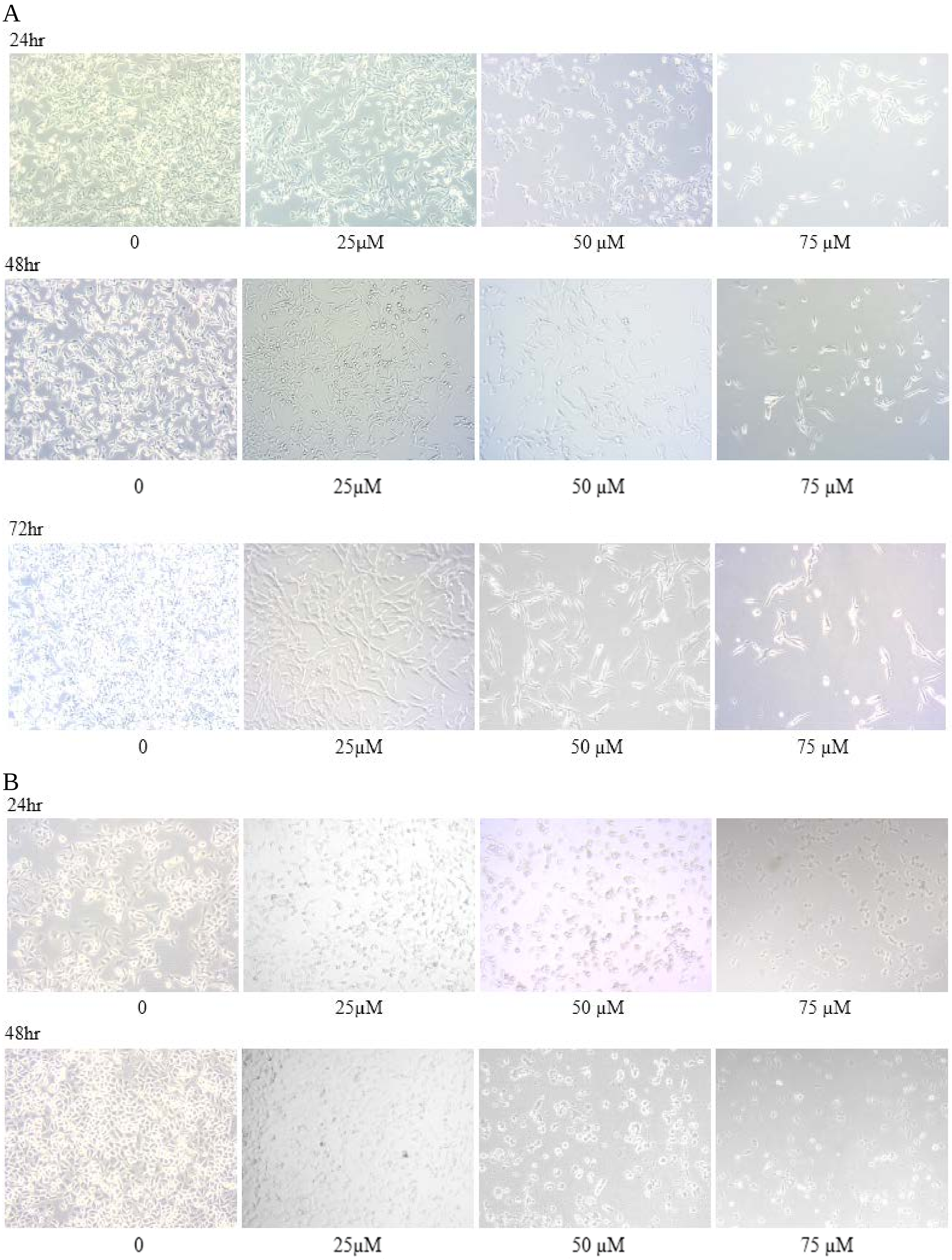

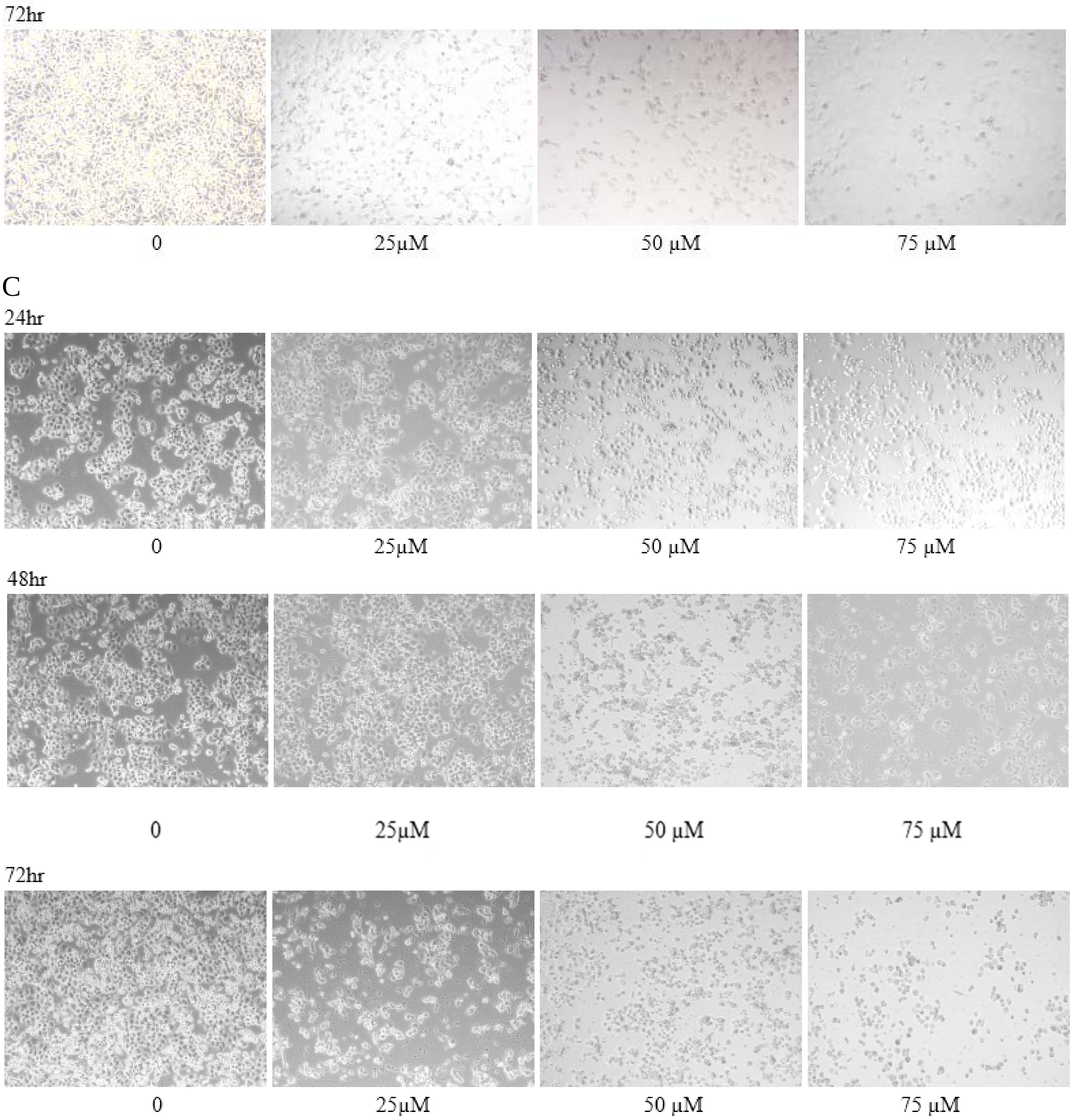

Supplementary Figure 1: Measurement of cell morphology by phase-contrast microscopy. **A**, **B** and **C** are photographical representation of the visual inhibition caused by increasing emodin treatment (0, 25, 50 and 75 μM) at 24, 48 and 72 h, as measured by phase-contrast for NCI-H-460 (Large cell carcinoma), A-549 (adenocarcinoma) and NCI-H-520 (squamous cell carcinoma) cell lines respectively.

A

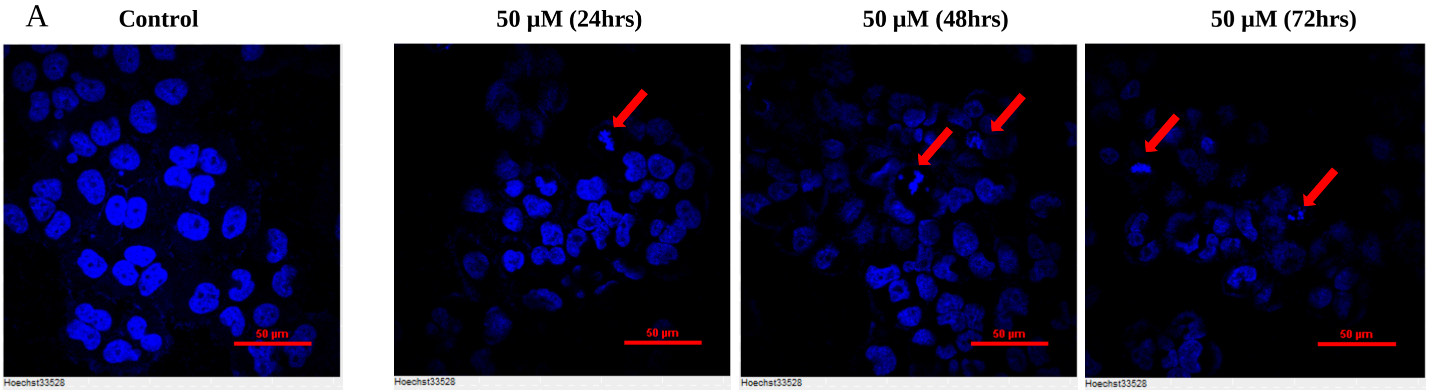

B

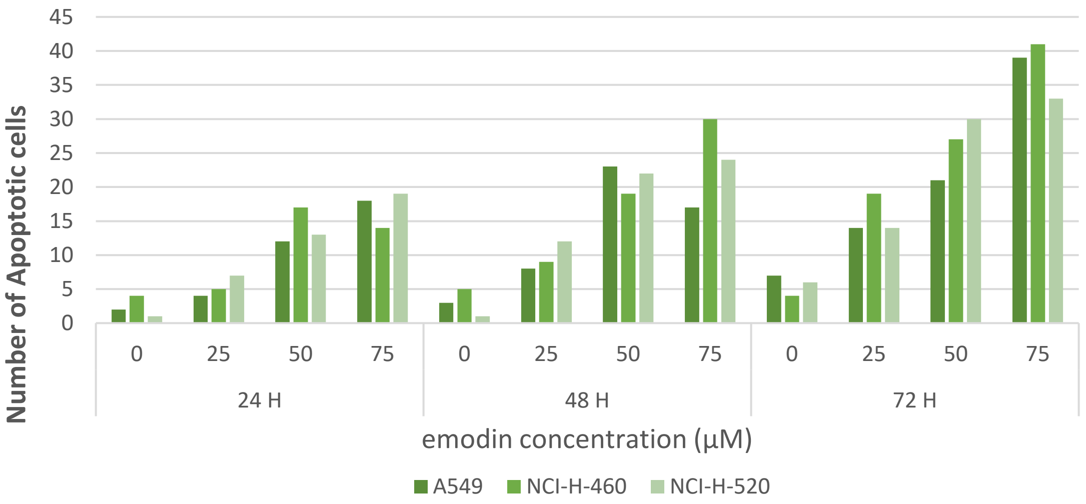

Supplementary Figure 2: **A**: Confocal microscopic images of NSCLC cell lines stained with Hoechst 33342 dye, showing disrupted nuclear morphology. **B**: Count of the number of apoptotic cells, in 50 frames for each sample as observed under the confocal microscope.

A

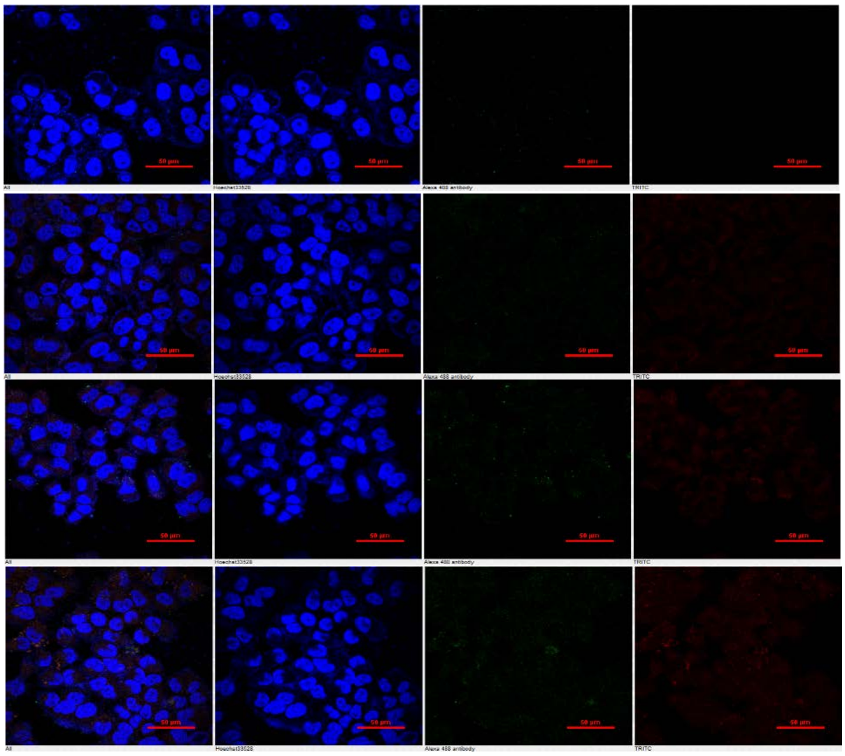

B

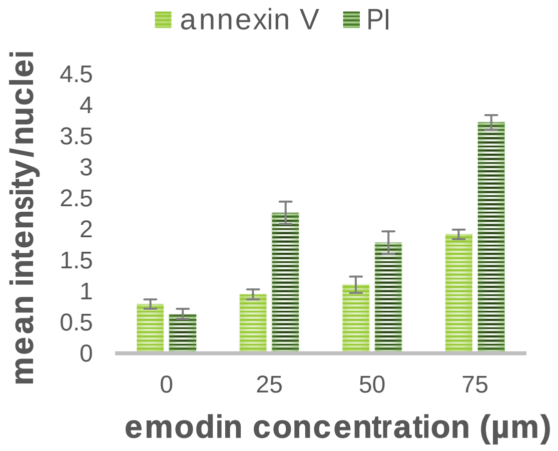

Supplementary Figure 3: **A**: Confocal microscopic images of NCI-H-520 cell line stained with Annexin V-FITC and PI dyes, showing increasing green fluorescence (Annexin positivity) and red fluorescence (PI positivity). **B**: Graphical representation of the mean fluorescent intensity/ nuclei indicating an increase in the fluorescence signal of apoptotic cells with increasing emodin treatment for 24h, as observed under the confocal microscope.

A

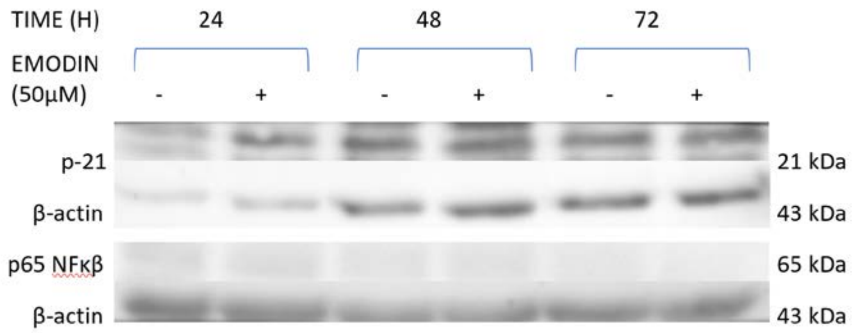

B

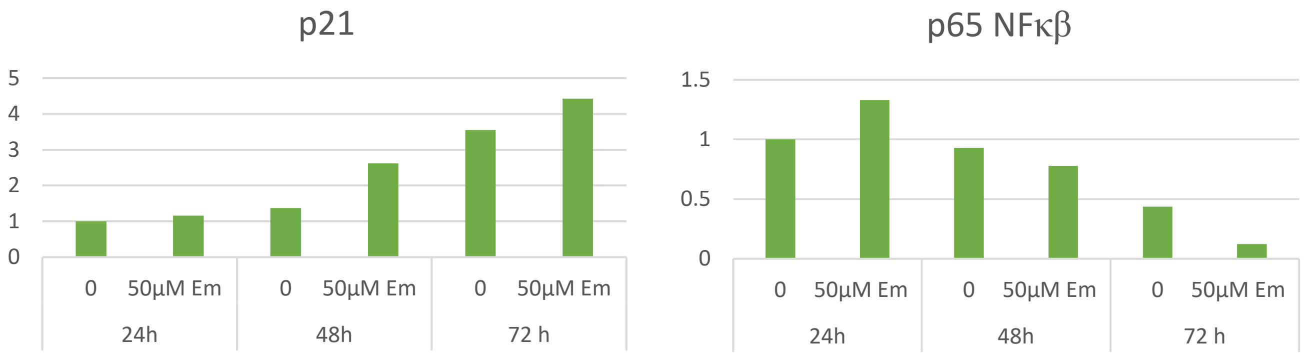

Supplementary Figure 4: **A:** Protein level expression profiles of p-21 and p65 NFκβ respectively. **B:** The graphical representation of the protein levels quantified by Image-J software. Data shown as mean ± S.E.M. of three independent experiments.

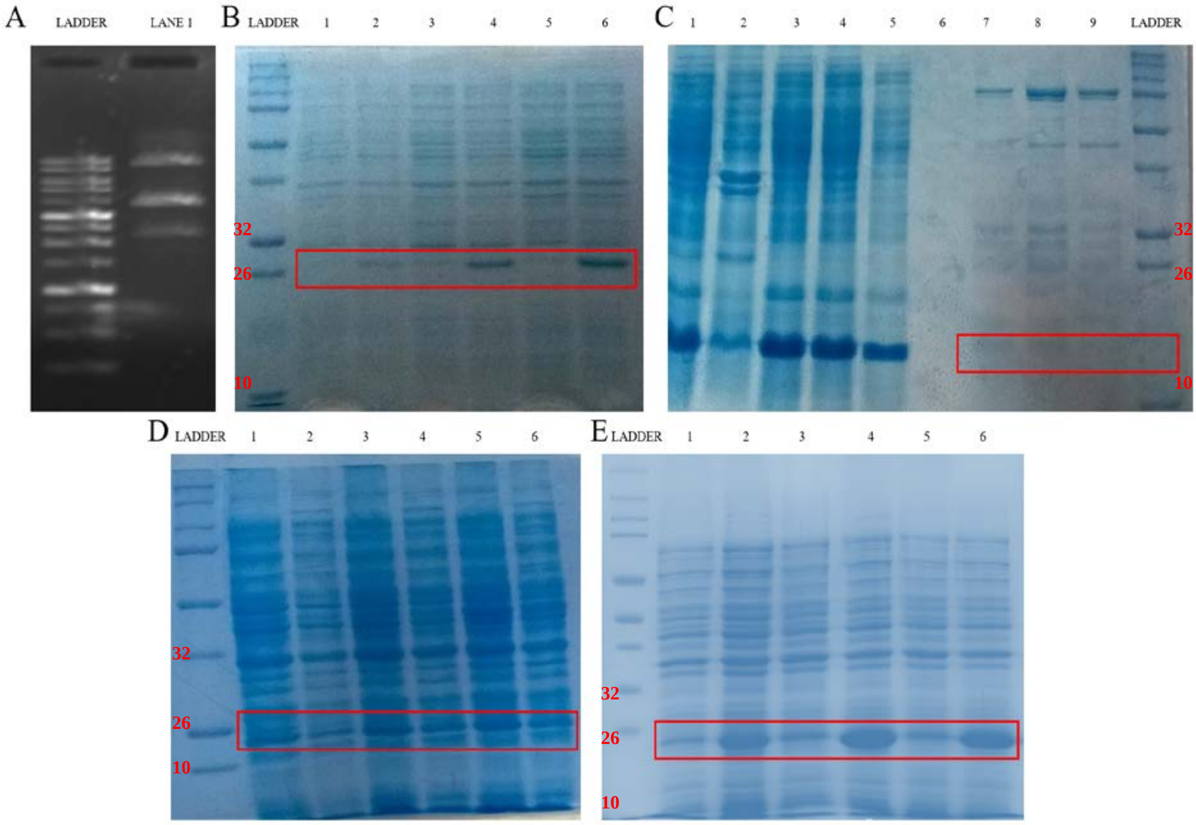

Supplementary Figure 5: Purification of MTH1 protein. **A**: MTH1 plasmid run on agarose gel for the determination of plasmid purity. Lane 1: MTH1 plasmid. **B**: BL-21 protein expression study on SDS page gel where Lane 1: un-induced @37°C for 1 h, Lane 2: IPTG induced @37°C for 1 h, Lane 3: un-induced @37°C for 3 h, Lane 4: IPTG induced @37°C for 3 h, Lane 5: un-induced @18°C overnight and Lane 6: IPTG induced @18°C overnight. **C**: the BL-21 protein purification study on SDS page gel where Lane 1: Total Cell Lysate, Lane 2: Cell Pellet, Lane 3: Lysate supernatant, Lane 4: Lysate Flow Through, Lane 5: Wash, Lane 6: Elution 1, Lane 7: Elution 2, Lane 8: Elution 3 and Lane 9: Elution 4. **D**: P-lyse protein expression study on SDS page gel where Lane 1: un-induced @37°C for 1 h, Lane 2: IPTG induced @37°C for 1 h, Lane 3: un-induced @37°C for 3 h, Lane 4: IPTG induced @37°C for 3 h, Lane 5: un-induced @18°C overnight and Lane 6: IPTG induced @18°C overnight. **E**: Rosetta protein expression study on SDS page gel where Lane 1: Lane 1: un-induced @37°C for 1 h, Lane 2: IPTG induced @37°C for 1 h, Lane 3: un-induced @37°C for 3 h, Lane 4: IPTG induced @37°C for 3 h, Lane 5: un-induced @18°C overnight and Lane 6: IPTG induced @18°C overnight. The data shown is representative of three independent experiments carried out under the same conditions.
